# Folding and Functionalizing DNA Origami: A Versatile Approach Using a Reactive Polyamine

**DOI:** 10.1021/jacs.4c12637

**Published:** 2025-01-27

**Authors:** Alejandro Postigo, Carlos Marcuello, William Verstraeten, Santiago Sarasa, Tobias Walther, Anabel Lostao, Kerstin Göpfrich, Jesús del Barrio, Silvia Hernández-Ainsa

**Affiliations:** † Instituto de Nanociencia y Materiales de Aragón (INMA), CSIC-Universidad de Zaragoza, Ed. I+D+i. Mariano Esquillor, Zaragoza 50018, Spain; ‡ Center for Molecular Biology of Heidelberg University (ZMBH), 9144 Heidelberg University, Im Neuenheimer Feld 329, 69120 Heidelberg, Germany; § Biophysical Engineering Group, Max Planck Institute for Medical Research, Jahnstr. 29, 69120 Heidelberg, Germany; ∥ Fundación ARAID, Av. Ranillas 1-D, 50018 Zaragoza, Spain; ⊥ Laboratorio de Microscopias Avanzadas (LMA), 16765 Universidad de Zaragoza, Ed. I+D+i. Mariano Esquillor, 50018 Zaragoza, Spain

## Abstract

DNA nanotechnology is a powerful synthetic approach to crafting diverse nanostructures through self-assembly. Chemical decoration of such nanostructures is often required to tailor their properties for specific applications. In this Letter, we introduce a pioneering method to direct the assembly and enable the functionalization of DNA nanostructures using an azide-bearing functional polyamine. We first demonstrate the successful polyamine-assisted folding of a scaffolded DNA origami nanostructure equipped with reactive azide groups. Leveraging this reactivity, we next showcase the decoration of the DNA origami via strain-promoted azide–alkyne cycloaddition with dibenzo­cyclooctyne-containing functional molecules. Specifically, we incorporate a fluorophore (Cy5), polyethylene glycol (PEG), and a hydrophobic phosphatidyl­ethanolamine (PE) tag to tailor the properties of our DNA origami nanostructures. Our approach is expected to streamline and reduce the cost of chemical customization of intricate DNA nanostructures, paving the way for enhanced versatility and applicability.

## Graphical abstract

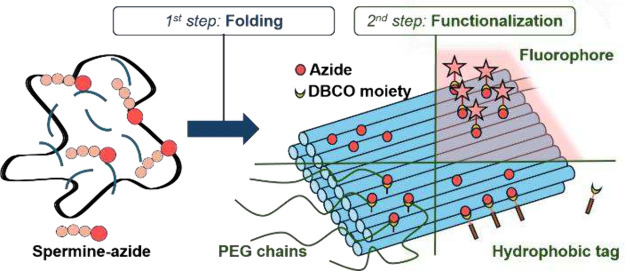

DNA nanotechnology stands out as a unique synthetic tool to produce tailored DNA nanostructures (DNS) boasting a wealth of applications across various fields. Through well-established assembly methodologies such as tile-assembly and scaffolded-origami, DNA strands effectively fold into nanostructures of predefined shapes and dimensions in a programmable manner.^1^ Indeed, programmable DNA assembly opens up numerous possibilities beyond the creation of soft nanoscopic DNA structures including the integration of specific labels and molecular tags. Synthetic DNA serves as a versatile framework for arranging a variety of molecules, including polymers, enzymes, and even larger inorganic particles, and significantly enhances the functionality of sometimes otherwise nonfunctional DNS.^2–4^ Indeed, in therapeutic delivery, bioimaging, and various biomedical-related applications, it becomes imperative to functionalize DNS with molecules like targeting tags, contrast agents, drugs, and stability-enhancing moieties.^5–7^ These modifications are typically accomplished through a two-step route, where specific strands containing reactive groups (e.g., azide, amine, or thiol groups) are introduced during the first step of the DNA assembly process. Subsequently, a bioconjugation reaction, such as strain promoted azide–alkyne cycloaddition (SPAAC), amidation via *N*-hydroxy­succinimide activated ester, or the thiol-maleimide Michael addition reaction, is employed to anchor the functional moiety to the DNS.^4^ Alternatively, these functional groups can first be coupled to reactive strands and then assembled to yield chemically-modified DNS.^8–10^ Although these strategies provide precise control over the placement of functional moieties, they require substantial synthetic effort and the use of reactive oligonucleotides and subsequent purification, which increase the cost and labor. This holds especially true when considering intricate designs such as DNA origami. For particular uses, achieving a sufficient degree of modification is prioritized over precise control in the position of functional tags on the DNS, with a greater emphasis on the efficient accessibility of functional DNA.^11–13^ This is for instance apparent when considering modifications that enhance stability in biological media^14^ or alter cellular uptake.^15^

Considering this, we introduce a novel strategy for folding and functionalizing DNA origami without the need for chemically-modified oligonucleotides. While precise control over functional positioning is not achievable, our approach allows for higher substitution levels of DNA origami in a versatile, straightforward, and cost-effective manner. We leverage recent advancements in DNA self-assembly facilitated by organic polyamines.^16–18^ While DNA self-assembly commonly relies on the use of magnesium (Mg^2+^), recent studies have shown that spermidine (Sp) is capable of assisting the folding of DNA nanoprisms^17^ and DNA tetrahedrons^19^ as well as larger DNA origami constructs.^16^ Other polyamines including spermine and putrescine have also shown to enable the assembly of tetrahedron-based structures.^20^ Here, we demonstrate that the azido spermine equivalent, SpAz (Figure 1), facilitates the folding of DNA origami in the absence of Mg^2+^. Moreover, the resulting DNS with azide-bearing reactivity is amenable to SPAAC, enabling the straightforward integration of tags with various characteristics into the DNA origami including the Cy5 fluorophore, a linear polyethylene glycol (PEG) polymer, and a lipid moiety, phosphatidyl­ethanolamine (PE), all containing a reactive dibenzo­cyclooctyne (DBCO) group as depicted in Figure 1b.

**Figure 1:**
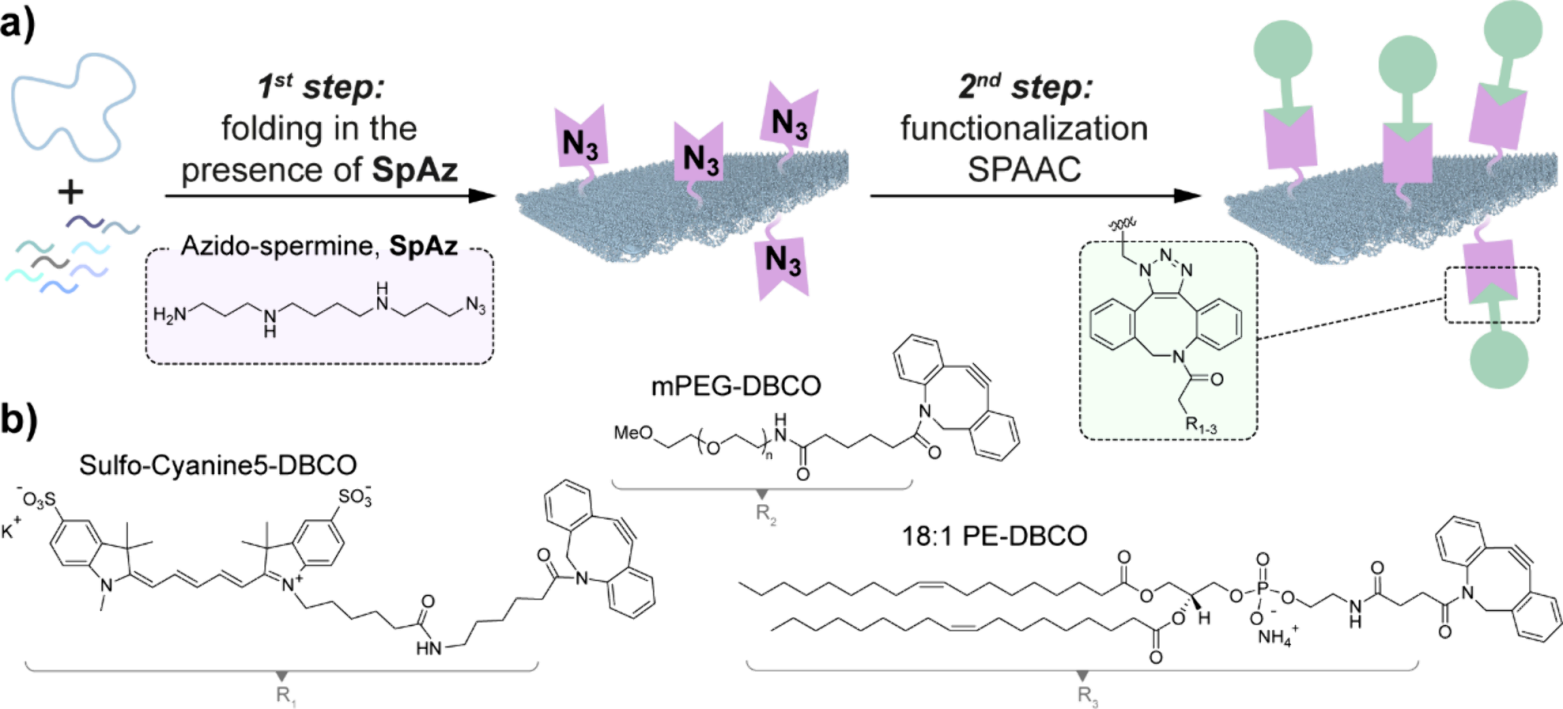
a) Schematic representation of the folding and functionalization strategy for DNA origami using spermine azide (SpAz). b) Chemical structure of DBCO containing moieties: i.e., mPEG, sulfo-Cyanine5 (Cy5), and 18:1 PE.

As a proof of principle, we designed a DNA origami nanostructure consisting of two layers of DNA arranged on a square lattice with approximate dimensions of 55 × 55 × 4 nm^3^ (Figure 2a).

**Figure 2:**
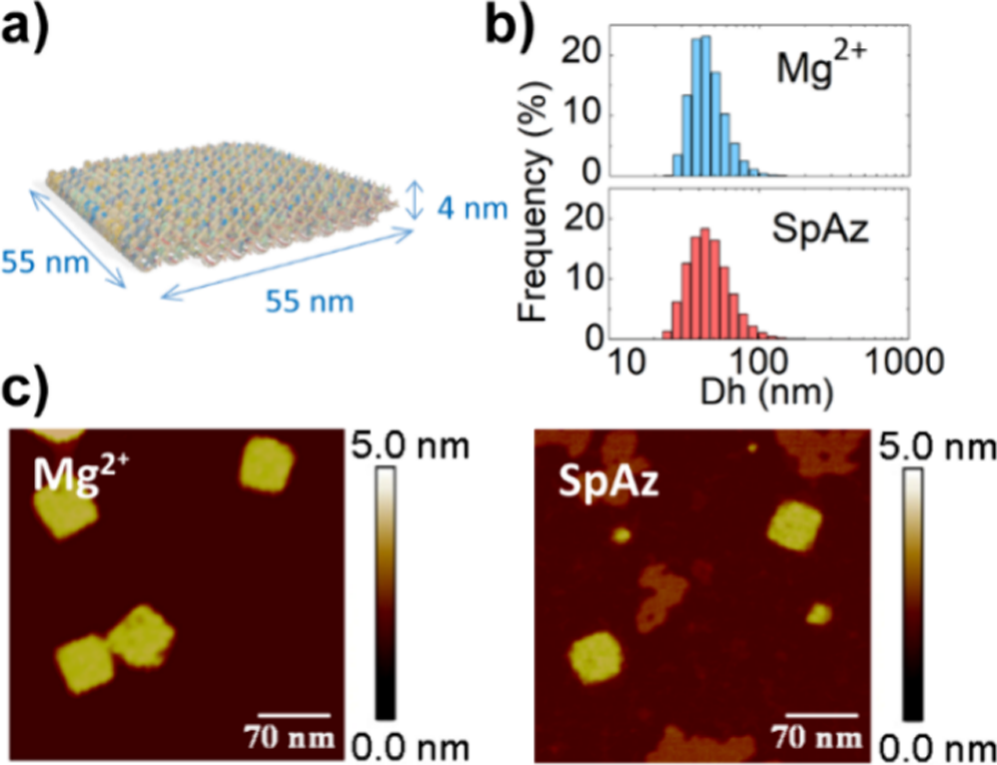
a) Schematic representation of the folded origami DNA nanostructure (DNS) by OxDNA. b) Hydrodynamic diameter (Dh) by DLS of Mg^2+^-folded DNS and SpAz-folded DNS. c) Representative AFM images of Mg^2+^-folded DNS and SpAz-folded DNS. Scan size is 350 nm × 350 nm.

The potential folding of the DNA origami was initially assessed within a gradient of different concentrations of SpAz in a Mg^2+^-free solution buffered with 1xTE (10 mM Tris-HCl, 1 mM EDTA) pH 8.2 followed by established thermal annealing protocols (see SI section 1.1). Agarose gel electrophoresis (GE) evidenced the appropriate folding, denoting the presence of a single band migrating in an analogous fashion to that corresponding to DNA origami folded in standard Mg^2+^-containing 1xTE buffer (Figure S4a). Specifically, at a scaffold concentration of 12 nM, the folding took place in the 250 to 300 μM range of SpAz (Figure S4b). Partial folding was observed at SpAz concentrations below 250 μM, whereas aggregation was dominant at 500 μM SpAz concentration. These values are consistent with previously reported Sp-folded DNA origami, maintaining a constant molar ratio of 1.5 between amine groups in SpAz and phosphate groups in DNA.^16^ Purification by size-exclusion chromatography (SEC)^16^ to remove the excess of staples did not affect the correct folding of the origami, as confirmed by GE (see Figure S4c). SpAz-folded DNS were further characterized via dynamic light scattering (DLS) (Figure 2b and Table S5), revealing a hydrodynamic diameter (Dh) of 47 ± 5 nm. This Dh value matches that of the DNS assembled in Mg^2+^ (46 ± 2 nm) as well as the expected size by design, evidencing the proper DNA origami assembly. The correct assembly of SpAz-folded DNS was further corroborated by atomic force microscopy (AFM), where well-formed square DNA origamis were observed, matching the obtained for DNS folded in standard Mg^2+^ solution (Figure 2c and Figure S8).

Having successfully established the SpAz-mediated DNS assembly, and aiming to show the versatility of our proposed approach, we attempted to modify DNS postassembly through SPAAC using functional moieties of diverse nature, including a Cy5 fluorophore, a linear PEG polymer, and a PE lipid derivative (Figure 1).

Initially, DNA origamis were functionalized with Cy5 fluorophores. The attachment of specific fluorophores to DNS is relevant for numerous applications in bioimaging and biosensing,^7,21–23^ as it enables the characterization and monitoring of DNS interactions with biological systems.^24–29^ Specifically, SpAz-folded DNS were reacted with sulfo Cy5-DBCO for 24 h at room temperature (rt), followed by SEC purification to remove any unreacted Cy5-DBCO. Additionally, two control functionalization reactions were conducted under similar conditions: (i) using spermidine (Sp) instead of SpAz, and (ii) using SpAz without DNS. Before SEC purification, a broad electronic absorbance band between 500 and 750 nm was detected in all cases (Figure 3a–c, full line spectra). After SEC purification, this band remained present when SpAz and DNS were used in the functionalization reaction (Figure 3a, dashed line) but disappeared entirely in the control samples (Figure 3b and c, dashed lines). These observations confirm the successful attachment of Cy5 to the SpAz-folded DNA origamis and demonstrate the effectiveness of our methodology in removing unreacted Cy5-DBCO.

**Figure 3:**
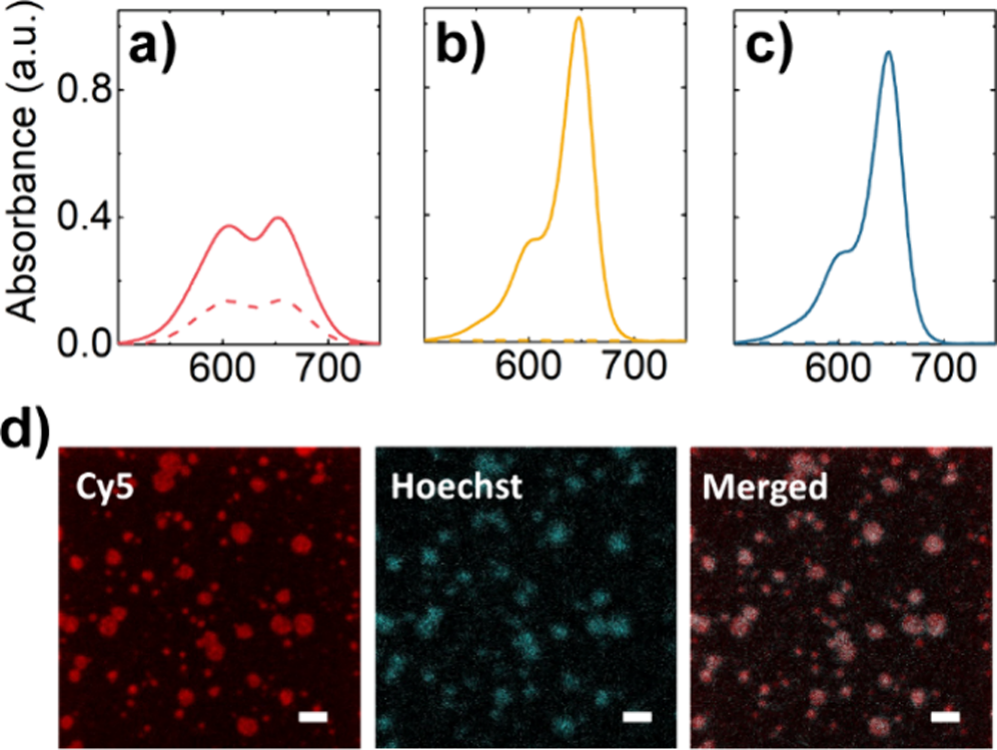
Spectra of Cy5-DBCO reacted with DNS samples before (full lines) and after (dashed lines) SEC purification: a) SpAz-folded DNS, b) Sp-folded DNS, and c) SpAz (no DNS). d) Confocal microscopy images of SpAz-folded DNS functionalized with Cy5-DBCO. Each channel (Cy5 and Hoechst) is shown in separate columns. Scale bar = 2 μm.

The number of Cy5 molecules per origami (after SEC purification), determined by absorbance considering Cy5 molar absorption coefficient, was estimated to be around 210, which represents approximately one Cy5 per 70 nucleotides (see SI, section 1.7 and Table S4). This substantial amount is challenging to achieve with other chemical decoration methods. Indeed, considering that DNA origami typically has about 200–250 staples, even if every staple could be modified, we would reach a similar range but at much higher cost. Appropriate Cy5-labeling of DNA origamis was further corroborated by GE using a gel documentation system monitoring in the Cy5 channel (Figure S6a) as well as by confocal microscopy, where highly fluorescent spots could be observed (Figure 3d) colocalizing with the signal of Hoechst 33342, a DNA staining agent. Cy5 spots were considerably brighter than the observed for a Mg^2+^-folded DNA origami modified with only 48 Cy5 fluorophores (see Figures S3c and S7a and Table S1). Thus, our SpAz-mediated modification approach presents a clear advantage in introducing a significant number of fluorophore moieties in a straightforward and reduced cost fashion.

Next, following a methodology analogous to that of the Cy5-functionalized DNS (*vide supra*), we attempted the attachment of PEG to the DNA origami. This modification is highly relevant for drug delivery applications. Indeed, PEG coating has been shown to influence the biodistribution of DNS and to alter the protein corona once DNS are introduced into biological media,^12,15,30^ thereby impacting their internalization capacities and biostability.^12,14,15,31^ The use of PEG-DBCO (10 kDa) for our functionalization strategy resulted in an increase in the Dh of the DNA origami, from 47 ± 5 nm (nonfunctionalized SpAz-folded DNS) to 74 ± 13 nm (after PEG functionalization and SEC purification). DNS folded with Sp and treated with PEG-DBCO showed no significant size change (Dh = 48 ± 7 nm), confirming that the azide group is key for the optimal modification and discarding any nonspecific interaction between PEG and the surface of the DNA origami (see Table S5). AFM topography images revealed a less defined square-shape morphology in PEG-modified DNS (Figure 4a) compared to SpAz-folded (Figure 4b) or Mg^2+^-folded DNS (Figure 4c), see also Figure S10. Interestingly, PEG functionalization affected the nanomechanical properties of DNS, leading to a softening of the nanostructure surface (Figure 4d–f, Table S6, and Figure S9). Indeed, the effective Young’s modulus of the PEG-functionalized DNS (421 ± 128 MPa) was significantly lower than that of SpAz-folded DNS (580 ± 198 MPa) and Mg^2+^-folded DNS (576 ± 137 MPa). This observation is in agreement with previous reported works regarding its elastic modulus and viscosity, associated with the soft nature and hydrated characteristics of the PEG coating.^32,33^ In addition, PEG modification resulted in an increase of roughness parameter (Ra) values (Figure S10). This effect could be attributed to the random distribution of PEG molecules on the DNA origami surface. Furthermore, the average height and relative volume increased from 3.5 ± 0.2 and 7718 ± 877 nm^3^ for SpAz-folded DNS to 5.0 ± 0.3 and 11978 ± 1860 nm^3^ for PEG-functionalized DNS (see Table S6 and Figure S9). Overall, this strategy introduces a new way for equipping DNA origamis with PEG coating and modifying their surface properties.

**Figure 4:**
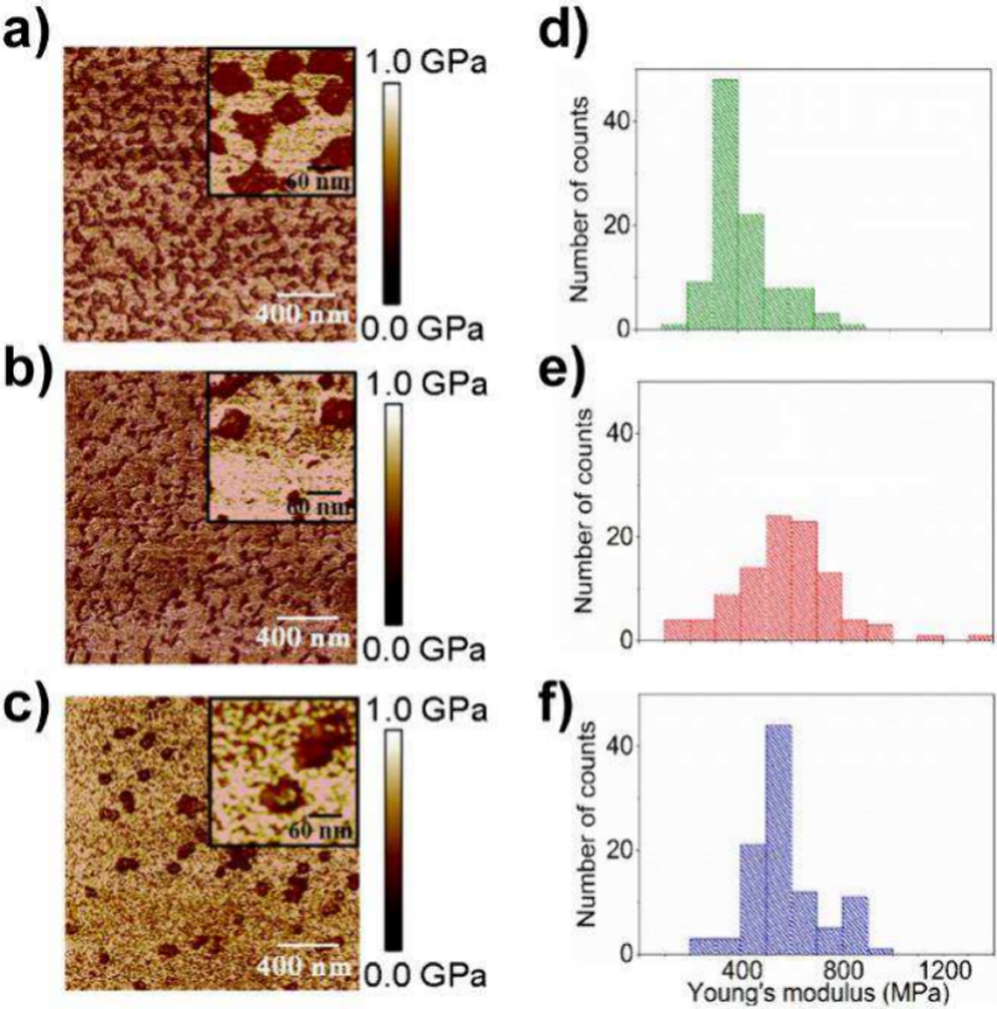
Representative Young’s modulus maps obtained from nanomechanical AFM images of: a) PEG-functionalized, b) SpAz-folded, and c) Mg^2+^-folded DNS. Scan size is 2 μm × 2 μm. The insets on the upper right side of each image depict the assessed features with higher resolution. Scan size of the insets is 300 nm × 300 nm. Histograms of the Young’s modulus values found for d) PEG-functionalized, e) SpAz-folded, and f) Mg^2+^-folded DNS.

Finally, the SPAAC functionalization of SpAz-folded DNS was exploited to mediate the attachment of DNS to giant unilamellar vesicles (GUVs), which serve as biomimetic models for cell membranes (Figure 5a).^34^ This approach holds significant promise in biophysical engineering for modulating and replicating natural cellular processes.^35–39^ Specifically, GUVs were composed of DOPC (2-((2,3 bis­(oleoyloxy)­propyl)­dimethyl­ammonio)­ethyl hydrogen phosphate) as the primary lipid, Liss Rhod PE (Lissamine Rhodamine B, 1,2-dioleoyl-*sn*-glycero-3-phosphoethanolamine-*N*-dibenzocyclooctyl) for fluorescent labeling (1% mol/mol), and 5% PE-DBCO to enable SPAAC-mediated functionalization. GUVs were incubated with SpAz-folded DNS containing Cy5-modified staples (see Table S1 and Figure S3c) for 24 h at rt, and the successful attachment was demonstrated by confocal microscopy, as evidenced by the colocalization of LissRhod-marked GUVs (in yellow) and Cy5-labeled DNS (in red) surrounding the GUVs (Figure 5b and Figure S12). Conversely, no attachment was observed in GUVs lacking PE-DBCO and incubated with SpAz-folded DNS, as evidenced by Cy5-labeled DNS being uniformly distributed throughout the external matrix (Figure 5c and Figure S13). Efficient attachment to PE-DBCO-containing GUVs has also been demonstrated for SpAz-folded DNS prefunctionalized via SPAAC with Cy5-DBCO (see Figure S16). This demonstrates that our method can be also exploited for multiple functionalization with different moieties.

**Figure 5:**
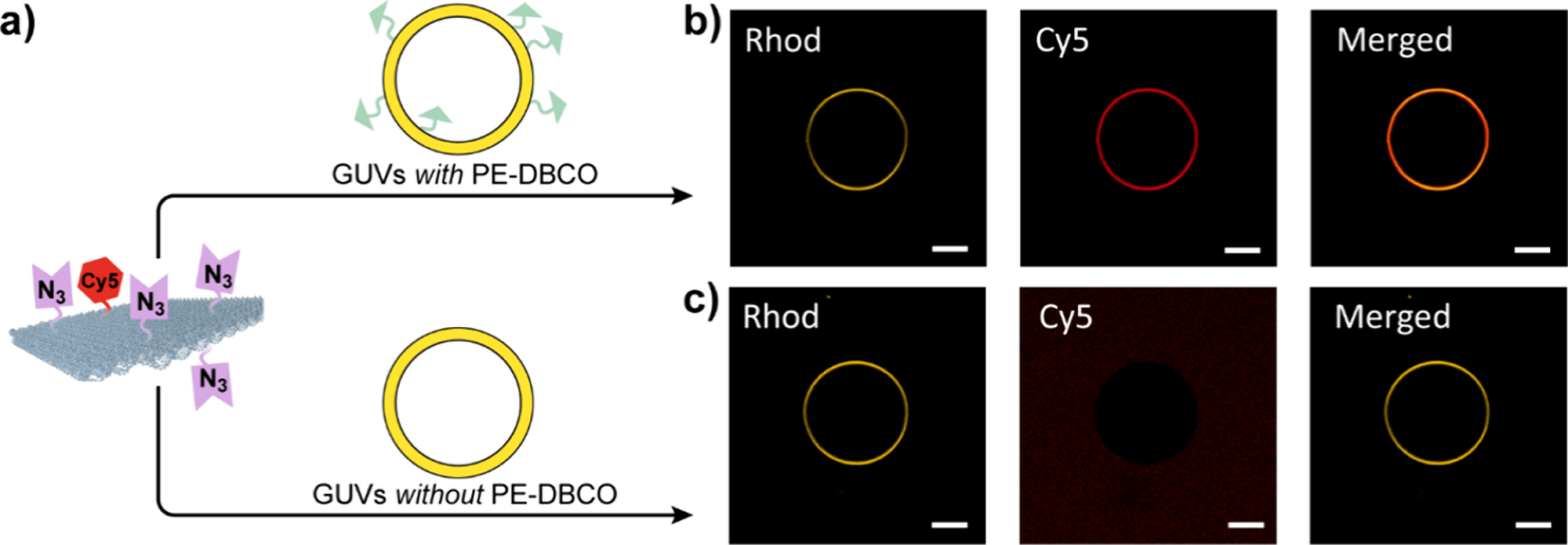
a) Scheme showing the attachment of SpAz-folded DNS to GUVs (Rhodamine-labeled) with PE-DBCO. Confocal images of b) GUVs with 5% PE-DBCO and c) without PE-DBCO incubated with SpAz-folded DNS (Cy5-labeled). Each channel (Rhodamine and Cy5) is shown in separate columns. Scale bar corresponds to 10 μm.

While a DNA origami nanostructure has been presented here to illustrate the viability of SpAz-mediated folding and functionalization of DNS, we have further validated our approach with other designs including a tetrahedron-based and 4-helix bundle DNS (see Figure S3 and data in the SI). Consequently, our strategy offers remarkable versatility, enabling the assembly and functionalization of a variety of DNS through a simple two-step process. Although controlling the precise placement of modifications is not achievable, its simplicity and adaptability make it particularly advantageous for applications where such spatial precision is not essential, providing higher degrees of substitution in a cost-effective fashion. The versatility of our approach is reflected in the variety of functional tags used, allowing customization of DNS for different purposes without compromising its overall structural integrity.

In summary, our study introduces a facile strategy to fold and subsequently functionalize DNA nanostructures by exploiting the reactive polyamine SpAz. We have successfully attached a variety of functional molecular tags to the DNS, with relevance to diverse fields, such as bioimaging, therapeutic delivery, and biomimetics. In essence, our straightforward and versatile method opens new opportunities for the preparation of functional DNS, thereby expanding the potential of DNA nanotechnology.

## Supplementary Material



## Notes

Methods, protocols, instrumentation, materials, and supplementary data (PDF)

## #

Biofisika Institute (CSIC, UPV/EHU), 48940 Leioa, Spain
