# Supplementary material for: Folding and Functionalizing DNA Origami: A Versatile Approach Using a Reactive Polyamine
Source: J Am Chem Soc. 2025 Jan 27;147(5):3919–24. doi: 10.1021/jacs.4c12637 (PMC12124716; doi:10.1021/jacs.4c12637)
Supplement: Supplementary file 1 [file ja4c12637_si_001.pdf]

Supporting Information for

Folding and Functionalizing DNA origami: a Versatile Approach Using a Reactive Polyamine

Alejandro Postigo,[a] Carlos Marcuello,[a],[b],‡ William Verstraeten,[c],[d] Santiago Sarasa,[a] Tobias Walther,[c],[d] Anabel Lostao[a],[b],[e] Kerstin Göpfrich,[c],[d] Jesús del Barrio[a]* and Silvia Hernández-Ainsa.[a],[e]*

[a] Instituto de Nanociencia y Materiales de Aragón (INMA), CSIC-Universidad de Zaragoza, Ed. I+D+i. Mariano Esquillor, 50018, Zaragoza, Spain.

[b] Laboratorio de Microscopias Avanzadas (LMA), Universidad de Zaragoza. Ed. I+D+i. Mariano Esquillor, 50018, Zaragoza, Spain

[c] Center for Molecular Biology of Heidelberg University (ZMBH), Heidelberg University, Im Neuenheimer Feld 329, 69120 Heidelberg, Germany

[d] Biophysical Engineering Group, Max Planck Institute for Medical Research, Jahnstr. 29, 69120 Heidelberg, Germany

[e] Fundación ARAID, Av. Ranillas 1-D, 50018, Zaragoza, Spain

‡ Current affiliation: Biofisika Institute (CSIC, UPV/EHU), 48940 Leioa, Spain

E-mails: jdb529@unizar.es, silviamh83@unizar.es

1. Methods, protocols, instrumentation and materials

1.1. DNS preparation

Assembly

DNA origami structures were prepared using the open source CaDNAno v0.2 software. A complete list of the DNA origami strands is provided in Table S1, while details of the design are shown in Figure 2a of the main text. Oligonucleotides were purchased from ThermoFisher and Macrogen (purification: standard desalting). The scaffold strand p7560 was obtained from Tilibit nanosystems (M1-31). Assembly of DNA origami structures involved the addition of strands in a fourfold excess relative to the p7560 scaffold, within a solution containing Spermine Azide (SpAz) (Iris Biotech, SNN1170) at different concentrations, buffered with TE pH 8.2 (Sigma Aldrich, T9285). For DNS folded with Mg^{2+}, origamis were assembled in a solution containing 12 mM $MgCl_2$ also buffered with TE. Assembly of DNS was conducted in a thermocycler (Bio-Rad T100), employing a 24-hour protocol involving heating to 85 °C and gradual cooling to 25 ºC, as detailed in Figure S1. Non-purified samples were stored at 4 °C until further use.

To assess the versatility of the developed modification strategy, other DNS designs were explored. Namely, a tetrahedron-based DNS[1] and 4-helix bundle DNS[2] were employed (Table S2 and S3, Figure S3). Oligonucleotides for these designs were procured from Macrogen (purification: standard desalting). Assembly of these DNS variants occurred at equimolar concentrations in a solution containing a range of SpAz concentrations buffered with TE (pH=8.2). For control experiments lacking SpAz: the 4-helix bundle DNS was assembled in 1x Phosphate Buffered Saline (PBS) (pH=7.4) and the tetrahedron-based DNS were folded in a solution containing 10 mM $MgCl_2$ buffered with TE (pH=8.2). As for the thermocycler protocols, tetrahedron-based DNS were assembled following previously published protocol[1] following these annealing steps: 95 °C for 5 min, 65 °C for 30 min, 50 °C for 30 min, 37 °C for 30 min, and 22 °C for 30 min. 4-helix bundle DNS were assembled in the thermocycler following a previous reported protocol[2] by heating to 85 °C for 5 min, followed by a gradual cooling from 85 to 65 °C in 20 steps (1°C per step, 5 min each step) and finally cooling from 65 to 25°C in 80 steps (0.5°C per step, 12 min for each step). Samples were stored at 4°C upon their use.

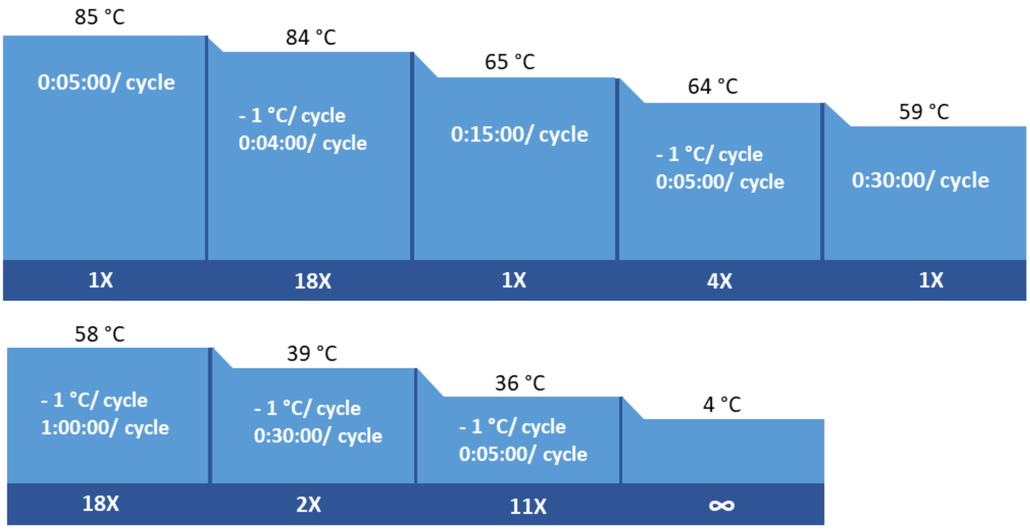

Figure S1. Thermal cycling protocol used for origami DNS. The initial temperatures of each step is shown at the top of the diagrams. At the bottom of the diagram the number of cycles per step is indicated. The temperature drops from cycle to cycle and the time per cycle is shown below the starting temperatures. After finishing the thermal processes at 25 °C, the structures were kept at 4 °C.

Purification

Mg^{2+}-folded DNS samples were suspended in 400 µL of 1 × TE, 3 mM $MgCl_2$ and the purification was carried out by spin filtration in a micro centrifuge (ThermoScientific, Sorvall Legend Micro 21R centrifuge) using 100 kDa cut-off filters (Amicon Ultra-0.5 Centrifugal Filter 100 kDa MWCO Millipore, UFC5100). Subsequently, the concentration of the origamis was assessed using UV-visible spectroscopy (UV-visible nanodrop spectrophotometer, DeNovix DS-11), and the $MgCl_2$ concentration was adjusted back to 12 mM. As reported by *A. Chopra et al*. in their supporting information section,[3] traditional methods of DNA origami purification were not suitable for polycation-folded structures. i.e, PEG filtration due to the high sodium chloride concentrations employed, which compete with polycations, spin columns due to the interaction of polycations with filter membranes, and gel extraction due to potential sample contamination, respectively. Therefore, for purification of SpAz-folded DNA origamis, size exclusion chromatography (SEC) was employed. To implement SEC, S300-HR sephacryl resin (Sigma Aldrich, S300HR) was initially equilibrated with MilliQ water, and 50% v/v slurry was prepared. The resin was packed into Pierce centrifugal columns (Thermo Fisher, 11894131) by spinning the columns at 800 relative centrifugal forces (rcf) for 3 minutes at 15 ºC. First, 400 µL of resin (800 µL of slurry) were packed into each column, followed by the addition of an extra 200 µL of resin (400 µL of slurry) to the column, resulting in a total of 600 µL of packed resin. Subsequently, a volume of 100 µL of unpurified SpAz-folded DNS was passed through two spin columns packed with 600 µL of resin and spun at 800 rcf for 3 minutes at 15 ºC. After purification, the concentration of the origami was determined by UV-Vis spectroscopy and corroborated by analyzing bands intensity in gel electrophoresis images. The recovery yield after purification was calculated by considering the initial number of moles of the non-purified DNS. The final recovery yield after SEC purification was in the range of 70-85%. This yield is in agreement with previous reports (see as examples *A. Chopra et al*.[3] and *L. Mallik, S. Dhakal et al*.[4])

For tetrahedron-based and 4-helix bundle DNS, purification was conducted via spin filtration, using 10 kDa cut-off filters (Amicon Ultra-0.5 Centrifugal Filter Unit, Millipore, UFC501096). Following purification, the concentration of the DNS was determined using UV-visible nanodrop.

1.2. DNS functionalization with Cy5.DBCO and PEG.DBCO

The DNS samples were reacted with sulfo Cy5.DBCO (Lumiprobe, 133F0) or mPEG.DBCO (IrisBiotech, RL-2540) at different molar ratios relative to the SpAz concentration employed for the folding. Specifically, for 25 nM DNS, 560 µM SpAz was required. This sample was mixed with either 560 µM of PEG.DBCO or 56 µM of Cy5.DBCO. Resulting in a mix of 12.5 nM DNS within 280 µM SpAz and 280 µM PEG.DBCO or 28 µM Cy5.DBCO. For control samples lacking PEG.DBCO or Cy5.DBCO, an equivalent volume of nuclease-free water was added. Incubation of the samples occurred over a 24-hour period at room temperature to facilitate the reaction. Following incubation, a portion of non-purified samples was extracted to serve as a comparison with purified samples. For the remaining samples, excess of unreacted Cy5.DBCO or PEG.DBCO was removed through an additional step of SEC purification. Specifically, the samples were passed through two spin columns packed with 600 µL of resin and spun at 800 rcf for 3 minutes at 15 ºC as depicted in Figure S2.
Note that for tetrahedron-based, and 4-helix bundle DNS, excess of unreacted Cy5.DBCO or PEG.DBCO was removed through an additional spin filtration process.

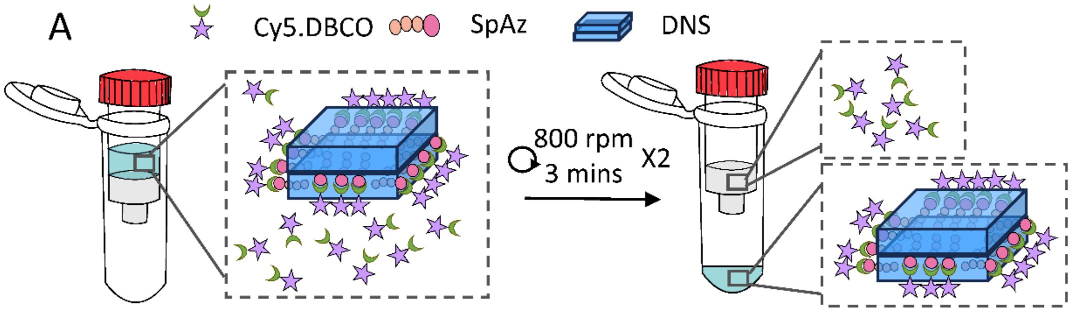

Figure S2. Scheme of Cy5.DBCO functionalized DNS purification by SEC to remove unreacted Cy5.DBCO.

1.3. DNS characterization by gel electrophoresis

The correct folding of the origami DNS upon SpAz concentration was assessed by agarose gel electrophoresis (AGE). For this purpose, DNS samples, with concentrations ranging from 50 to 75 ng/µL, and supplemented with gel loading dye (New England Biolabs, B7024S) were loaded onto a 1% (w/v) agarose gel. Electrophoresis was conducted for 45 minutes at 80 V in 0.5 × Tris-borate-EDTA (TBE) buffer pH 8.3 (ThermoFisher, BP1335) supplemented with 11 mM $MgCl_2$. For subsequent visualization, the gels were stained in GelRed (Biotium, 41003) and subjected to visualization under UV light transillumination (BioRad, GelDoc Go Imaging System).

The correct folding of tetrahedron-based and 4-helix bundle DNS at various SpAz concentrations was evaluated using polyacrylamide gel electrophoresis (PAGE) due to their lower molecular weight. For this analysis, 20 ng of each DNA sample was loaded onto the gel. The samples underwent electrophoresis for 1 h at 100 V, employing either a 10% polyacrylamide gel for 4-helix bundle DNS or a 6% polyacrylamide gel for tetrahedron-based DNS. The gel was immersed in a solution containing 11 mM $MgCl_2$

buffered with 1x Tris-Acetate-EDTA (TAE) pH 8.3 (Thermo Fisher, 10628403). To serve as a reference, a 100 bp DNA ladder or 1 kb DNA ladder (New England Biolabs, N3231 and N3232) were run alongside the samples.

1.4. DNS characterization by Dynamic Light Scattering (DLS)

The hydrodynamic diameters (Dh) of DNS were determined using DLS. DNS concentrations were settled to 10 nM and transferred to a low-volume disposable cuvette (Malvern, ZEN0040). The samples were analysed at 25 ºC using a Malvern analytical Zetasizer Nano ZS instrument. Reported values represent the average of 10 measurements, each comprising 5 reads per run. Values in number are provided. For tetrahedron-based and 4-helix bundle DNS, concentration was settled to 2 µM.

1.5. DNS characterization by Atomic Force Microspy (AFM)

AFM imaging

Each sample was prepared by incubating a 6 µL droplet containing 10 nM DNS, folded either with Mg^{2+} or SpAz and with or without PEG (where the Mg^{2+} concentration was adjusted to 12 mM in every sample to strengthen the electrostatic adsorption to the substrate) onto a freshly cleaved mica piece for 10 minutes. Subsequently, the substrates were gently washed three times with filtered Milli-Q water, and excess liquid with immediate removed of using a tissue. The sample was then air-dried for 3 minutes under a gentle flow of nitrogen and imaged immediately after preparation.

AFM measurements were performed with a MultiMode 8 system equipped with Nanoscope V Controller (Bruker) operating with PeakForce-Tapping mode (PF-TM) in air.[5] PF-TM brings the cantilever tip into contact with the sample surface, applies continuous force-distance curves, and sets the tip-sample feedback by the peak force.[6] This method provides higher control over the tip-sample interaction forces, thereby increasing resolution by directly measuring this interaction at each image pixel. These advantages make PF-TM suitable for unraveling the morphology of soft samples such as the oligonucleotide secondary structure of DNA strands, aided by reconstruction studies.[7] The images were obtained using ScanAsyst-air probes carrying pyramidal tips providing a nominal final radius of 2 nm (Bruker Probes). Ultrasharp tips minimize the detrimental effects of AFM tip convolution broadening, inherent during scanning.[8] Prior to AFM imaging, the deflection sensitivity (nm/V) and the spring constant (nN/nm) of the cantilevers were properly calibrated by conducting at least three force-distance curves on a stiff sapphire surface, obtaining the average value of the slope from the retraction curves and through the thermal tune method,[9] respectively. The gathered spring constants of the chosen AFM probes were close to the nominal value of 0.4 N/m provided by the manufacturer. Several images from different scan areas were recorded for each sample with scan sizes of 2 µm x 2 µm, 1 µm x 1 µm and 600 nm x 600 nm. Continuous force curves were conducted at constant frequencies of 1 kHz, allowing acquisition rates of 0.8-0.9 Hz. AFM images were recorded with a resolution of 512 x 512 data points.

The roughness arithmetic average (Ra) parameter was calculated from the height deviations rendered on top of the visualized DNA features (1):

$$R_a = \frac{1}{N}\sum_{i=1}^{N}\left|Z_j\right| \quad (1)$$

being *N* the population size, *Z* the absolute height value measured, and *j* the number of image pixels considered, respectively.

Raw AFM image analysis and data processing were performed using WSxM,[10] Gwyddion,[11] and NanoScope Analysis (Bruker) software tools. The volumes of individual particles were estimated by setting a defined height threshold mask, thus eliminating interferences by background subtraction. These volumes are relative, since AFM provides greater values than the real ones due to the tip widening effect.[7] Nonetheless, measuring all the samples in the same conditions makes feasible the comparison. All AFM images were treated consistently following the same process and selecting the same settings to prevent scattering on height, volume, and roughness (Ra) values of the analysed samples based on data handling. Mean values and standard deviation (SD) were calculated from 50 values (N = 50). Histograms were plotted defining bin sizes of 0.10 nm, 500.0 nm^3 and 0.02 nm for height, volume, and Ra respectively.

Young's modulus determination

The same AFM setup and software were employed to estimate the Young's modulus of Mg-folded DNS, SpAz-folded DNS and PEG functionalized DNS. PeakForce Quantitative Nanomechanical Mapping (PF-QNM) mode (Bruker) was operated in air once the tip apex radius of the AFM probe was calibrated through convolution analysis of a scanned titanium roughness reference surface. PF-QNM precisely controls the extension of indentation exerted by the AFM tip, measuring the maximum force required to maintain contact, which is suitable to spatially resolving the elasticity deformation properties of soft matter systems.[12] The Derjaguin-Müller-Toporov (DMT) model was utilized to ascertain the Young's modulus of the samples according to the geometry of the tip at loaded forces of 0.9 nN. The DMT model is valid for sharp hemispherical nanoindenters, taking into account the long-range Van der Waals interactions exerted between the tip and the external sample surface[13] and relies on the loaded force of the nanoindenter (2):

$$F - F_{adh} = \frac{4}{3}E^{*}\sqrt{R(d-d_0)^3} \quad (2)$$

Where F is the applied force, F_{adh} is the tip-sample adhesion force, E^* refers to the apparent Young's modulus value, R is associated to the contact radius between both surfaces, and (d-d_0) is the indentation depth. Then, the Young's modulus of DNS samples (E_{DNA}) can be calculated through the following expression (3):

$$E^{*} = \left(\frac{1-\upsilon_t^2}{E_t} + \frac{1-\upsilon_{DNA}^2}{E_{DNA}}\right)^{-1} \quad (3)$$

Where E_t is the Young's modulus of the AFM tip material, υ_t and υ_{DNA} are the Poisson's ratio values of the tip and DNA sample, respectively. In this study, a Poisson's ratio of 0.5 is considered for the DNS samples, as per to previously reported works,[14] which is consistent with the consideration of DNA as a perfectly incompressible isotropic material susceptible to be elastically deformed at small strains.

The Young's modulus of Mg^{2+}-folded DNS, SpAz-folded DNS and PEG functionalized DNS was determined by analyzing 100 values (N = 100). Mean values and standard deviation (SD) were calculated and histograms were plotted using a bin size of 100.0 MPa.

Statistical Analysis

For AFM measurements, data recorded from height, volume, Ra and Young's modulus of Mg-folded DNS, SpAz-folded DNS and PEG functionalized DNS were compared by one-way analysis of variance (ANOVA) combined with the Tukey-Kramer post hoc and Pearson tests. Meaningful differences were assumed with significant levels greater than 95% ($p<0.05$). Analysis was done using OriginPro 8.5 software tool. Box plots of data with the statistical significance are gathered in Figure S9.

1.6. DNS Characterization by Transmission Electron Microscopy

A 10 µL drop of each sample was placed on Parafilm in a Petri dish. Freshly incandescently discharged (30 s, 15 mA) carbon-coated 200-mesh copper grids (Agar Scientific Supplies) were incubated over the sample drops for 5 min. Excess solution was removed by applying a filter paper to the edge of the grid and then stained with 2% uranyl acetate in water for 1 min. Excess solution was removed by contacting the edge of the grid with filter paper. The grids were then stored in an EM grid box in a desiccator for future observation using a Tecnai T20 microscope (Thermo Fisher Scientific) at 200 kV.

1.7. Absorbance measurements and determination of Cy5 molecules per DNS

The estimation of the amount of Cy5 molecules per DNS was done by UV-Vis spectrophotometry recording the absorbance related to Cy5 spectra.

Non-purified and SEC-purified samples (see section 1.2.) were transferred to a quartz micro cuvette (Hellma™, 105-202-85-40) and placed into a CaryBio 100 UV-vis spectrophotometer (Varian). Absorbance readings were taken within the 500 to 800 nm range, with the maximum absorbance intensity recorded at 650 nm. Data analysis was performed using OriginPro 2016 software. Initially, the concentration of Cy5.DBCO in DNS within SpAz sample post-purification was determined using the Beer-Lambert model (4)

$$A = c * l * \varepsilon \quad (4)$$

Here, *A* represents the absorbance value at 650 nm, *c* denotes the Cy5.DBCO concentration, *l* signifies the cuvette's light pass width (1 cm for Hellma™ 105-202-85-40), and ε is the extinction coefficient, set at ~270000 $L \cdot mol^{-1} \cdot cm^{-1}$ according to the producer (Lumiprobe).

This calculation yielded the estimation of the Cy5 concentration. Subsequently, the Cy5 concentration was normalized by the DNS concentration, and the final ratio of Cy5 to base pairs (bps) was determined (see Table S4).

For the other DNS, a similar process was conducted but with different initial concentrations. Specifically, 1 µM DNS folded with 50 µM SpAz/Sp were incubated with 5 µM Cy5.DBCO.

1.8. Visualization of cy5-modified DNS by confocal microscopy

Prior to visualization, Cy5-functionalized DNS were incubated for 10 minutes with Hoechst 34580 (2 mg/mL in MilliQ water, Sigma Aldrich) at a 1:1000 dilution to achieve DNA staining. Subsequently, the samples were placed in an untreated, custom-built observation chamber, allowing them to adhere to the glass slide.

For comparative analysis of fluorescence intensity, the SpAz-folded DNS functionalised with Cy5 was compared to a typical fluorescent DNA origami. This DNA origami was assembled in a buffer containing TE and 14mM $MgCl_2$, and was fluorescently labelled at 48 positions by mixing it with a 2-fold excess of Cy5-tagged single-stranded DNA (Biomers, purification: HPLC) complementary to 48 extensions protruding from one face of the DNS (Table S1) . Schematic design information is provided in Figure S3c.

Imaging was performed using an LSM 900 Zeiss confocal fluorescence microscope (Carl Zeiss AG). The pinhole aperture was set to one Airy Unit, and all experiments were conducted at r.t. Images were acquired using a 63x oil immersion objective (63x/1.2 W korr, Carl Zeiss AG). Post-acquisition image processing and analysis were carried out using the ZEN 3.4 software.

1.9. DNS attachment to GUVs

GUVs preparation

Giant unilamellar vesicles (GUVs) were generated using the electroformation method facilitated by a VesiclePrepPro device (Nanion Technologies GmbH). Two distinct types of GUVs were produced for the experiments detailed in the main text: PE.DBCO-containing GUVs and PE.DBCO-lacking GUVs.

PE.DBCO-containing GUVs were comprised of 94 % DOPC (1,2-dioleoyl-sn-glycero-3-phosphocholine, Avanti Polar Lipids, 850375C), 1 % 18:1 LissRhod PE (1,2-dioleoyl-sn-glycero-3-phosphoethanolamine-N-(lissamine rhodamine B sulfonyl), Avanti Polar Lipids, 810150C) and 5% 18:1 PE.DBCO (1,2-dioleoyl-sn-glycero-3-phosphoethanolamine-N-dibenzocyclooctyl, Avanti Polar Lipids, 870129C). Conversely, control PE.DBCO-lacking GUVs were composed of 99% DOPC and 1% LissRhod PE.

To prepare the GUVs, 40 µL of a 2 mM lipid mixture in $CHCl_3$ was fast and homogeneously spread onto the conductive side of an indium tin oxide (ITO) coated glass coverslide (Visiontek Systems Ltd) using a cover slide. The lipid-coated ITO slide was then subjected to vacuum conditions for a minimum of 30 min to ensure complete evaporation of the $CHCl_3$. Subsequently, an 18 mm diameter rubber ring was positioned on the lipid-coated ITO slide and filled with 275 µL of 200mM sucrose (Sigma-Aldrich, S8501). A sealed chamber was created by placing a second ITO slide on top of the rubber ring. The assembled electroformation chamber was inserted into the VesiclePrepPro. A programmable AC-current with an amplitude of 3 V and a frequency of 5 Hz was applied across the ITO slides for 2 h at 37°C. GUVs were promptly collected after formation, diluted 1:6 with 200 mM glucose, and stored at 4°C for up to 2 days.

Incubation of GUVs with DNS

Fluorescently labelled DNS were used for potential visualization by confocal microscopy. To this end, prior to assembly with SpAz in TE and purification from excess staples, the

DNS were fluorescently labelled at 48 positions as explained in section 2.1. See Table S1 and Figure S3c for further information about design.

An incubation mixture of GUVs and DNS was prepared by combining 9 µL Cy5-tagged DNS at a concentration of 25 nM within 590 µM SpAz, 10 µL of diluted GUVs (with or without PE.DBCO), 1 µL 10 mM $MgCl_2$ and 950 mM NaCl to ensure sufficient salt concentration for DNS attachment to GUVs,[15] and 2 µL of 165 mM glucose to maintain osmolarity. The mixture was then incubated for 24 h at r.t. and visualized thereafter.

We further demonstrate that GUVs anchoring was also achieved using SpAz-folded DNS fluorescently labelled via SPAAC reaction with Cy5-DBCO. For this purpose, SpAz-folded DNS were reacted for 24 hours with Cy5-DBCO (1 Cy5-DBCO molecule per 40 SpAz molecules added for DNS folding). The resulting Cy5-DBCO functionalized SpAz-folded DNS structures were then purified by SEC, as described in section 1.2, prior to their incubation with GUVs.

To evaluate the versatility of the developed strategy for attaching DNS to GUVs, an atto-488 labelled tetrahedron-based DNS was utilized (see Figure S3b). The incubation with GUVs was done by mixing 9 µL of atto-488 labeled tetrahedron DNS at a concentration of 5 µM (folded with 500 µM SpAz), 1 µL of $MgCl_2$ 40 mM, 2 µL of 850 mM glucose, and 10 µL of diluted GUVs (with or without PE-DBCO). The mixture was then incubated for 24 hours at room temperature and subsequently visualized.

Confocal imaging

GUVs were imaged in a custom-built observation chamber, which was coated with 50 mg/mL Polyvinyl-alcohol (Sigma–Aldrich) for 5 minutes to prevent the GUVs from fusing with the glass coverslip.

Imaging was conducted using an LSM 900 Zeiss confocal fluorescence microscope (Carl Zeiss AG). The pinhole aperture was set to one Airy Unit, and experiments were performed at r.t. Images were acquired using a 20× objective (Plan-Apochromat 20×/0.8 M27, Carl Zeiss AG). Subsequent image processing and analysis were carried out using the ZEN 3.4 software.

2. Supplementary data

2.1. Oligonucleotide sequences

Table S1. Sequences of the staples used to prepare origami DNS. For 48 extension DNS, core strands were substituted by extension strands which hold an overhang indicated in blue. Note that Cy5-labeled strand complementary to overhang is indicated at the last row.

Core1	CAGTTTGGAACAAGAGTCCACTAAATCAAAATACAAACTTATGGGAT
Core2	TTCACCGCCTGGCCCTGAGAGAGCACCAGTGAGCGGAGTTATCGGTT
Core3	AATCGCCATATTTAACAACGCCGTATAAAGGCGCCTGTTATCATTC
Core4	GCTTAATTGCTCATTTTTGATTCAAAAGTCATTGCCTGAGAGT
Core5	TCAAAAAGATTTCAGAAGCTCGATGAATAAAATTCGCATTAAA
Core6	AATGTTTAGACTGCCAGAGATTTTTTAGATTGACCGTAATGGG
Core7	ATGAAAATAGGATTTTTTAAAATTAAGATAGCTTAGATTAAG
Core8	AATACCACATTATTCATCAGATGGGCGCATTCGCCATTCAGGC
Core9	AAGAAACAATAGTTAAGCGTAGATTTCAAAAGAAGATGATGA
Core10	AGACCAGGCGCGAGGACAGTTCTAAGTTCTATGATACCGACAG
Core11	AAGGTAAATACATTCAACGAATTGAGGTTATTAATTTTAAAA
Core12	CGCGAAACAAAACCCCCAGAAGCAACTGTGCTTGTTACCTCGA
Core13	TCAGTAGCGAACCATCGACGAACGAAATATCTGGTCAGTTGG
Core14	CGGCTACAGAGGCATCGGAGGGTACCGAGTGAGCTAACTCACA
Core15	ACCCTCAGAAAACCGCCTTTTACATTACTGATAGCCCTAAAA
Core16	CGCAGTCTCTTCATTAAAATTAACCGTACATTTTGACGCTCA
Core17	CGCCACCCTCAGAACCGCCACGCGGGGTTAAAGCGAAGCTAGGGC
Core18	AAATCAAGTTTTTTGGCGGGGAAAAGAGGGTTTGAAAGTA
Core19	GGGCGATGGCCCACTAAAGGGAAGTTGCTCAGAGAAGGAT
Core20	TCCAACGTCAAAGGGCTGGTGGTTACAGCCCTTCTTTCCA
Core21	GAGCCCCCGATTTAGAGCTTGAGGTCGAGGTACTCAGG
Core22	GAGAAAGGCGTGAACCAAC
Core23	CTGTTTGAGAAAAACCCCACCACCCTCATTTT
Core24	CCCTTATATTAAAGAACAATAGGA
Core25	ACCACCACGCACGTATATAAACAGTAAGCGTC
Core26	GCTGGCAAAGAGCGGGCGGGGTCAGAGTGTAC
Core27	TTTGCCCCGCGTATTGGGAATTGCAAAAAAAG
Core28	CGCGTACTGGCCACCGAGTAAAAG
Core29	TTTGACGAACCCGCCGCTGAAACAGATATAAG
Core30	TTAGAATCGTGTAGCGTCCTCAAGTACCAGGC
Core31	GGCGGTTTAGCAGGCGTTTTGTCGCATAGTTA
Core32	TTTCTTTTTTGCAGCAATTTTCTGACAACGCC
Core33	GAGAAGTGTTGATTAGTGATATTCCACCACCC
Core34	ATTTTAGAGAAGAACTAGGAGGTTACCAGAGC
Core35	AATCGGCCAACATACGACGCATAAGTTAAAGG
Core36	CGGGAAACCCTGGGGTGTTGCGCCTCAGCAGC
Core37	ATACTTCTTTTTTATACGTTCCAGTTAATGCC
Core38	CCTGAGTACAGGAACGTGATACAGGTGCCTTG
Core39	TTCCACACAACGCGCGAATCTCCAGAATAATA
Core40	GTGTAAAGCTGTCGTGTTTAATTGGAGAATAG
Core41	AGGAAAAAACACGACCCACCGGAATTTAGCGT
Core42	CCAGAACACAACAGAGTTAGCGTTGCATTTTC
Core43	TGAAATTGCTGACCTCTTAAACGGACCTAAAA
Core44	TTCGTAATGGGCTTAAGACTAAAGAAAACACT
Core45	CACCAGTCCGCTCATGCTCAGAGCACAAACAA
Core46	ATTCTGGCATATTACCGAACCACCGAGGCAGG
Core47	TCCGAACTTTATCCGCTGCAGGGACCGATATA
Core48	AGTAAACACATGGTCACGTCACCCGACAATGA
Core49	TGGCTATTAAACAGAGAAGGCCGGATTAAAGG
Core50	AAGAATACGCCTGCAACAAAATCAGAGCCATT
Core51	CGCATTTCCCAAAATACGAAATCCGGCGCAGA
Core52	GGCACGAACTCGCCCTGAGATTTGCTGACCAA
Core53	AGAAGATAAGTCTTTAAGTTTGCCCCAGAGCC
Core54	TTAACACCGTGGCACATTTCATCGTGCCATCT
Core55	ATGTCCCGACATAAATAGGCACCAGTAAAATA
Core56	TATTTACGTATAGGGGAATACACTACTTTTTC
Core57	CCTCAAATATCTTTAGCCAGCGCCTAAAGGTG
Core58	AATGAAAAGAGCCGTCGTCACAATGACACCAC
Core59	CTGAATTGACGCCAGGAACCGGATTCATTCAG
Core60	ATTCATGCAAACGACGGCTGACCTAAACACCA
Core61	TCTAAAATATCAAACCAATTATTCAAACGTCA
Core62	ATAGATTAATCTAAAGACCGACTTCCAGTAGC
Core63	GTTGGGTATCAACCTTCGGAACGAGCGACCTG
Core64	ACGTTGTAGCACGACTGAACCGAATATCATCG
Core65	TCGTATTAGAAGGAGCATACCCAATCTTACCG
Core66	AAGTATTATATCAGATAAGGAAACCAGATAGC
Core67	GGATGTGCGCCAGCTTTGGCTCATACGTTAAT
Core68	CTCTTCGCAAACCAGGATTGTGAATATTACAG
Core69	AACCACCAAATCCTTTTACATACAAAAGACAA
Core70	TTCCTGATGACTTTACAACGCAAACAATAGAA
Core71	GCACTCCATGCAAGGCAAAGCTGCATTCATTA
Core72	GGTGCCGGTATTACGCCTGACGAGTCATCAAG
Core73	GGGTTAGAATGAATATCAGAGAGAAATAACAT

Core74	ATTGTTTGGGGAGAAAACAAAGTCGGGAGAAT
Core75	CAGTATCGTGTGAGCGGCATAGTAACGACGAT
Core76	CCGTGCATGTGGGAACGCAGATACTTTGCAAA
Core77	AACGTCAGACCTACCAAATAGCTAAAGAACTG
Core78	TTTACATCGATTATACAAAGTAAGCGAGGAAA
Core79	ACATTAAAGCCTCAGGAAAAATCTTATACCAG
Core80	GATTCTCCCTGCCAGTAACAACATTTACCTTA
Core81	AATTATTCCATTTGAATTTATCCCCTACAATT
Core82	GAATACCAACATAAATTTTGCCAGAACGAGCG
Core83	TTCCTGTAGAAGATTGATTGAATCAGAATGAC
Core84	GAACGCCAAACGTTAAGTCCAATAACCCTGAC
Core85	AACAATTTATTTCAATACAGAGAGTAACCCAC
Core86	GAAACAGTAGTTACAACATTAGACAGAGGGTA
Core87	AAAAACAGGCCAGCTTTTTACCAGAGAGCAAC
Core88	AAATTGTATCAAAAATGAGAGGCTATAACGCC
Core89	CCTTAGAACTGAGAGATTTTAGCGATTTTCAT
Core90	TTCTGTAAGGTTGGGTGGCTTATCATAGCAAG
Core91	GGTTGATAATGCCGGATCGAGCTTAGGTCAGG
Core92	GTAAAACTCTACAAAGGCCCGAAACCTTTTGA
Core93	TCATAGGTTCCTTGAAGCACCCAGAATCCAAA
Core94	CCGGCTTAATCGTCGCCCAACGCTTTACAAAA
Core95	ATAAATTAATCAGAAAAGAAAACGCCCCTCAA
Core96	TGAGAGATAGCATGTCAGGTCTTTCTGCGGAA
Core97	AAACTTTTACCGACCGTCTTTCCTTTATCAAC
Core98	CAAATCCAAGAATAAAGAGCATGTAAATAATA
Core99	TGATATTCTAAAAATTCTAAAGTATGATTCCC
Core100	AAGGCCGGATGCAATGTGCTGTAGGTTTGACC
Core101	TTTGAAATTCAAATATCCGTTTTTAACCTCCC
Core102	GTTAAATAATCGCAAGCGCGCCCACGGTATTC
Core103	CGCAAGGAAACCGTTCACTCCAACCAAAGCGA
Core104	TATTTTAAAGACAGTCTAATTGCTGACTTCAA
Core105	TCTTACCAAACATGTACCAGACGA
Core106	GCCTGTTTCCAGTAATAAAGTACC
Core107	GCGGGAGAACATCCAAGCGAGCTG
Core108	ACCAAAAAAATTAGCATAACCTGT
Core109	AAAGCTAAAATGTGTAGG
Core110	AGAGGCATTTTCGAGAGTATCATAACAAGAAAGAAACCA
Core111	AATAGTAGTAGCATTAAGCCTTTAATAACAGTCGGTGTCT
Core112	ACAGGCAAGGCAAAGCATTATGATAGATTTACTCAACAT
Core113	CGACAATAAACAACATTGCAGAACCCAACGCTTTTAATGG
Core114	GACAAAAGGTAAAGTAGTCCTGATGCGTTATAAGGC
Core115	AAAAGGTGGCATCAATCATTCCATTTTATTACTCATACAA
Core116	TTAGCTATATTTTCAGAACGAGCCCTGTACCTCATA
Core117	AATAGATAAATTCTGTATTTAGGC
Core118	TCCCATCCTCTAATATAAGAGACT
Core119	AATTCTGCTTTGGGGCTAAATCAT
Core120	ATTAGATAATGGTCAAAAATTAAGCAATA
Core121	CAAGAACGCAAGCAAGATTTTAGTTATCAAAA
Core122	ATCAATATCATTACACAAAGATTAACCT
Core123	GGAAGTTTGGAAGCAATAGGTAAATATATCTG
Core124	GTTTTAAGTACCTTAAATCACCTATTTT
Core125	TCATCGAGAAGGTATTAAGACCTAAACAACAGTA
Core126	CGTAGGAAATCGGCTGTGTGATAAATACAAAT
Core127	CAAATCAGTAATTTACCACCGGAATAGAAAAA
Core128	ATTAGAGAATATGCAATTTAGAACATACTTTT
Core129	TAAGAGGTGAATATAACCTGAGTATCGGTTGT
Core130	GACTTGCGGCTATTTTAACATAGCTTACATTT
Core131	TAAGAACAATCTTATATTAATTTTAATG
Core132	ACCAGACCAACAGTTCAGCTGAATGTGAGCCC
Core133	ATATCGCAAAAATCAATCATAAATATTT
Core134	AAGATTAGTTGGAGGTTTAGTGAATTTAATTTCA
Core135	TTATCCTGGCGAGGCGCTACCTTTACGCGAGA
Core136	TCTTTCCAATATAGAATATATAACTGCTGATG
Core137	CATAAATCGTTTTAATGAGGGTAGCATCAATA
Core138	TATTATAGAAGAGGAAGCTATCAGGGGTGAGA
Core139	TAAGAAACCAGCCTTTTACCTGAGTCAGGTTT
Core140	TAAACAGGGAAGCGAATCGCGAGTACCT
Core141	ATGCTTTAAACCCTCGTCAGCCTGGATTCTCA
Core142	TCGTCATAAATAGCAATTCGCCCCGTCG
Core143	AAAAACAGCCATATTATTACCTTTTAATTTTC
Core144	TAACTGAAGAGCCTAACAATATATAACCTTGC
Core145	AAAAACCAAAATATTCTATAAGCATGTACCCC
Core146	AGAAGTTTTGGATAGCTATTTTGTCGGTAATC
Core147	AAGAATTGGAAATAGCTATCAAAAAACAAAGA
Core148	ATTGAGCTTTAAGATTCTGAACATCATA
Core149	ACTATCATTTGGGAAGAAGCAATATTCATATC
Core150	AAAAGGACTAACGGTTGAGGGCGCTTCT
Core151	AAGCCCTTGCTAATATACAGTAACCAGAGGCG
Core152	CGAACAAACACCCTGACAATAACGATTGCTTT
Core153	AAAACGAAATTACGAGAGTAACAAGTCTGGCC
Core154	GTAGAAAGCAACTAATAAACGGCGACCAATAG
Core155	GCATGATTGTAGAAAAGCCCGAACGAAGGTTA
Core156	CGCAATATAAAAGAAAACAATACAACTA
Core157	TCAGGACGAACGTAACGATTTTGAATACATAA
Core158	TGCGATTGCTTGCCCAGCTGGAGTCACG
Core159	TTTAATTTCAAAGTAAATTGGCGATCGCAAGCTTTCTCAGGAG

Core160	GTTAGCAAACAAGACTCCTTTTGCGGTTATTTGCACGTAAAA
Core161	GCAACATAATAACGGAGGAATTATTAATGGAA
Core162	GGAATAAGGTTACCAGGATGGCAATAATCCTG
Core163	TGAATAAGTTAAGAACTCCGGCACGACGACGA
Core164	GAACGAGTCTTTAATCCAAAGCGCCATCGTAA
Core165	AAGGGCGATTGACGGACTCAATCACCACCAGC
Core166	AATTCATCACCGTCCATCACCGTCAGTA
Core167	CCCAAATCTACTTAGCATGAGAGCCGCTGACA
Core168	AGTAATCCATAAGGTAAGTGTTCTAATC
Core169	TGAATTATATGGTTTAGAGCACTATCGACAAC
Core170	TGGGAATTTTTATTTTAATAGATAGGATTTAG
Core171	CGGTCAATTTGACAAGGTTTTCCCCGAAAGGG
Core172	CTTTGAAAATAGGCTGGCCAGTGCGTGCGGGC
Core173	CCAATGAACAGAATCAATGCGCGAGGCAGATT
Core174	ACCATTAAGCGCGTGACAATAAAGGGAC
Core175	CTCCATGTCACTACGACATGACCTCTTCTTTC
Core176	CCTGATAGCAAAAGCCTTGAATGTAATG
Core177	CAGACTGTCCATTAGCGTGAGGCGTTGCTGAA
Core178	GGTCATAGAGAGCCAGCAGTGCCACAGCAGCA
Core179	CGAAAGAGAATTGTGTACCCCGCTCCTTAGTG
Core180	CATCTTTGGTACAACGGGAGTGACGGTTGTGA
Core181	ACCACCGGCCGCCACCGAAATACCTTGTAGCA
Core182	TTTCATAGCCACCAGCCAGCCTCACTTG
Core183	CGTAATGCCTGAGGCTTCATTGCTCGGCCCAA
Core184	ATGAGGAGCGGGATTAGCTGTGCATAAA
Core185	TCAGAGCCATCAAAATAGTAATAATTTTTGAA
Core186	CGCCGCCACCCCCTTAATAGAACCGAAAGCGT
Core187	CCGCTTTTAGTTTCCACTGGTTGGTCGGCTGA
Core188	GAAAGACAGCTTTGAGGCTACGTGCGTCGGTG
Core189	ATAAATCCGAATTTACATCAGTGAATGGTTGC
Core190	TCAGACGCTTTTGAGTACGCCTTCCTCG
Core191	TTCGGTCGACGTTGAAGGGGCCGAAGGAGAGA
Core192	CAACAACAGGAGCCCCAGCTGGGTGGTT
Core193	AAACAGCTTGATGCTTTCGCTGCCCGCAACAGCT
Core194	ATACATGGATTGGCCTTAATAACAATTGCAAC
Core195	TGGTAATAGCATTGACCAAACTATGGTAATAT
Core196	GCTCCAAACATCGCCCAGCCGGAATTCCTGTG
Core197	TATCAGCTTACCGATAGCCTAATGAGCTCGAA
Core198	CCCTGCCTCTATTATTCGCTTAATGCGCCGCT
Core199	AGTAACACTGAGACGTCACGCACGTGGC
Core200	ATTTTTTCATCTAAAGAAACGGGCAGGAGATC
Core201	AAAGGAATAAATGAAGCGGTCGGCAAAAT
Core202	TTAAGAGGGTGCCCGTAACGTGCTAGAATCCT
Core203	TAGGATTAAGTTTTAAAGCTAAACTTAAAGGG
Core204	GACGTTAGCAACTAAAGGCGCCAGCATTAATG
Core205	TTTGCTAAACAGTTTCAGACGGGCTTTCCAGT
Core206	TATAGCCCTATCACCGTGCCGTAAAGCACT
Core207	GGATAAGCCCTCAGATCACCC
Core208	GCGTAACGCCTCAGAGGTCTATCA
Core209	TGTAGCAGCAAGCCCGTGGAC
Core210	AGGTTTAGTACCGCCATGCCGTCGGCCGGCGATGCGCGTA
Core211	CAGGGATATTCCACAGCCGAAATCCACGCTGG
Core212	ACCCATGTACCGTAACGTCACCAGGAATAGCCCGAGATA
Core117extension	GACTTGCGGCTATTTTAACATAGCTTACATTTTATCGCCTATGGATCTT
Core118extension	TAAGAACAATCTTATATTAATTTTAATGTATCGCCTATGGATCTT
Core119extension	ACCAGACCAACAGTTCAGCTGAATGTGAGCCCTATCGCCTATGGATCTT
Core120extension	ATATCGCAAAAATCAATCATAAATATTTTATCGCCTATGGATCTT
Core127extension	TAAGAAACCAGCCTTTTACCTGAGTCAGGTTTTATCGCCTATGGATCTT
Core128extension	TAAACAGGGAAGCGAATCGCGAGTACCTTATCGCCTATGGATCTT
Core129extension	ATGCTTTAAACCCTCGTCAGCCTGGATTCTCATATCGCCTATGGATCTT
Core130extension	TCGTCATAAATAGCAATTCGCCCCGTCGTATCGCCTATGGATCTT
Core133extension	AAAAACAGCCATATTATTACCTTTTAATTTTCTATCGCCTATGGATCTT
Core134extension	TAACTGAAGAGCCTAACAATATATAACCTTGCTATCGCCTATGGATCTT
Core135extension	AAAAACCAAAATATTCTATAAGCATGTACCCCTATCGCCTATGGATCTT
Core136extension	AGAAGTTTTGGATAGCTATTTTGTCGGTAATCTATCGCCTATGGATCTT
Core137extension	AAGAATTGGAAATAGCTATCAAAAAACAAAGATATCGCCTATGGATCTT
Core138extension	ATTGAGCTTTAAGATTCTGAACATCATATATCGCCTATGGATCTT
Core139extension	ACTATCATTTGGGAAGAAGCAATATTCATATCTATCGCCTATGGATCTT
Core140extension	AAAAGGACTAACGGTTGAGGGCGCTTCTTATCGCCTATGGATCTT
Core143extension	AAGCCCTTGCTAATATACAGTAACCAGAGGCGTATCGCCTATGGATCTT
Core144extension	CGAACAAACACCCTGACAATAACGATTGCTTTTATCGCCTATGGATCTT
Core145extension	AAAACGAAATTACGAGAGTAACAAGTCTGGCCTATCGCCTATGGATCTT
Core146extension	GTAGAAAGCAACTAATAAACGGCGACCAATAGTATCGCCTATGGATCTT
Core147extension	GCATGATTGTAGAAAAGCCCGAACGAAGGTTATATCGCCTATGGATCTT
Core148extension	CGCAATATAAAAGAAAACAATACAACTATATCGCCTATGGATCTT
Core149extension	TCAGGACGAACGTAACGATTTTGAATACATAATATCGCCTATGGATCTT
Core150extension	TGCGATTGCTTGCCCAGCTGGAGTCACGTATCGCCTATGGATCTT
Core153extension	GCAACATAATAACGGAGGAATTATTAATGGAATATCGCCTATGGATCTT
Core154extension	GGAATAAGGTTACCAGGATGGCAATAATCCTGTATCGCCTATGGATCTT
Core155extension	TGAATAAGTTAAGAACTCCGGCACGACGACGATATCGCCTATGGATCTT
Core156extension	GAACGAGTCTTTAATCCAAAGCGCCATCGTAATATCGCCTATGGATCTT
Core157extension	AAGGGCGATTGACGGACTCAATCACCACCAGCTATCGCCTATGGATCTT
Core158extension	AATTCATCACCGTCCATCACCGTCAGTATATCGCCTATGGATCTT
Core159extension	CCCAAATCTACTTAGCATGAGAGCCGCTGACATATCGCCTATGGATCTT
Core160extension	AGTAATCCATAAGGTAAGTGTTCTAATCTATCGCCTATGGATCTT
Core163extension	TGAATTATATGGTTTAGAGCACTATCGACAACTATCGCCTATGGATCTT

Core164extension	TGGGAATTTTTATTTTAATAGATAGGATTTAGTATCGCCTATGGATCTT
Core165extension	CGGTCAATTTGACAAGGTTTTCCCCGAAAGGGTATCGCCTATGGATCTT
Core166extension	CTTTGAAAATAGGCTGGCCAGTGCGTGCGGGCTATCGCCTATGGATCTT
Core167extension	CCAATGAACAGAATCAATGCGCGAGGCAGATTTATCGCCTATGGATCTT
Core168extension	ACCATTAAGCGCGTGACAATAAAGGGACTATCGCCTATGGATCTT
Core169extension	CTCCATGTCACTACGACATGACCTCTTCTTTCTATCGCCTATGGATCTT
Core170extension	CCTGATAGCAAAAGCCTTGAATGTAATGTATCGCCTATGGATCTT
Core173extension	CAGACTGTCCATTAGCGTGAGGCGTTGCTGAATATCGCCTATGGATCTT
Core174extension	GGTCATAGAGAGCCAGCAGTGCCACAGCAGCATATCGCCTATGGATCTT
Core175extension	CGAAAGAGAATTGTGTACCCCGCTCCTTAGTGTATCGCCTATGGATCTT
Core176extension	CATCTTTGGTACAACGGGAGTGACGGTTGTGATATCGCCTATGGATCTT
Core183extension	TCAGAGCCATCAAAATAGTAATAATTTTTGAATATCGCCTATGGATCTT
Core184extension	CGCCGCCACCCCCTTAATAGAACCGAAAGCGTTATCGCCTATGGATCTT
Core185extension	CCGCTTTTAGTTTCCACTGGTTGGTCGGCTGATATCGCCTATGGATCTT
Core186extension	GAAAGACAGCTTTGAGGCTACGTGCGTCGGTGTATCGCCTATGGATCTT
Cy5-strand	/Cy5/AAGATCCATAGGCGATA

Table S2. Sequences of the oligonucleotides used to prepare 4-helix bundle DNS.

4-helix1	AAAACGCTAAGCCACCTTTAGATCCAAA
4-helix2	AAAGTGCAGAGGCACGAATTCCCTCAAA
4-helix3	GAGGGAATTCGCCCGTCTCGACCGCACGACCTGGCTTAGCGT
4-helix4	GGATCTAAAGGACTTCTATCAAAGACGAGGCTGCCTCTGCAC
4-helix5	GGTCGTGCGGAAAAAATGATAGAAGT
4-helix6	GCCTCGTCTTAAAAAATCGAGACGGG

Table S3. Sequences of the oligonucleotides used to prepare tetrahedron-based DNS. For atto488-labeled DNS, strand Tet1 was substituted by Tet1-atto488. Note that atto488 labeled strand is indicated.

Tet1	ACATTCCTAAGTCTGAAACATTACAGCTTGCTACACGAGAAGAGCCGCCATAGTA
Tet2	TTCAGACTTAGGAATGTGCTTCCCACGTAGTGTCGTTTGTATTGGACCCTCGCAT
Tet3	TATCACCAGGCAGTTGACAGTGTAGCAAGCTGTAATAGATGCGAGGGTCCAATAC
Tet4	TCAACTGCCTGGTGATAAAACGACACTACGTGGGAATCTACTATGGCGGCTCTTC
Tet1-atto488	/atto488/ACATTCCTAAGTCTGAAACATTACAGCTTGCTACACGAGAAGAGCCGCCATAGTA

2.2. DNS designs

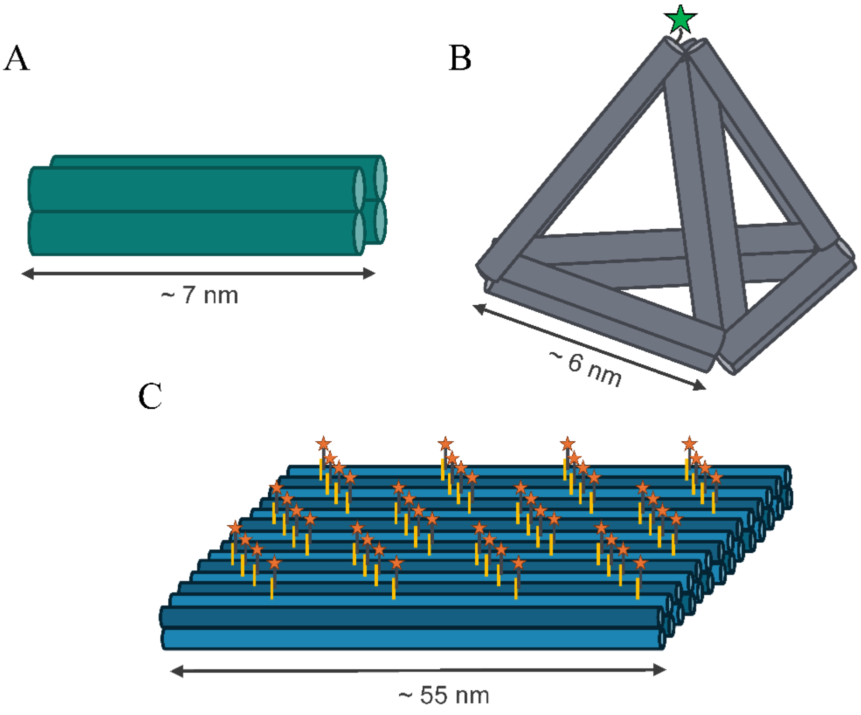

Figure S3. DNS schematic design layout. A) 4-helix bundle DNS. B) atto488-labeled tetrahedron-based DNS, with the atto488 residue represented in green. C) Cy5-labeled origami DNS, with DNA strand overhangs represented in orange and Cy5 residues in red.

2.3. Characterization of the DNS by gel electrophoretic mobility shift assay (EMSA)

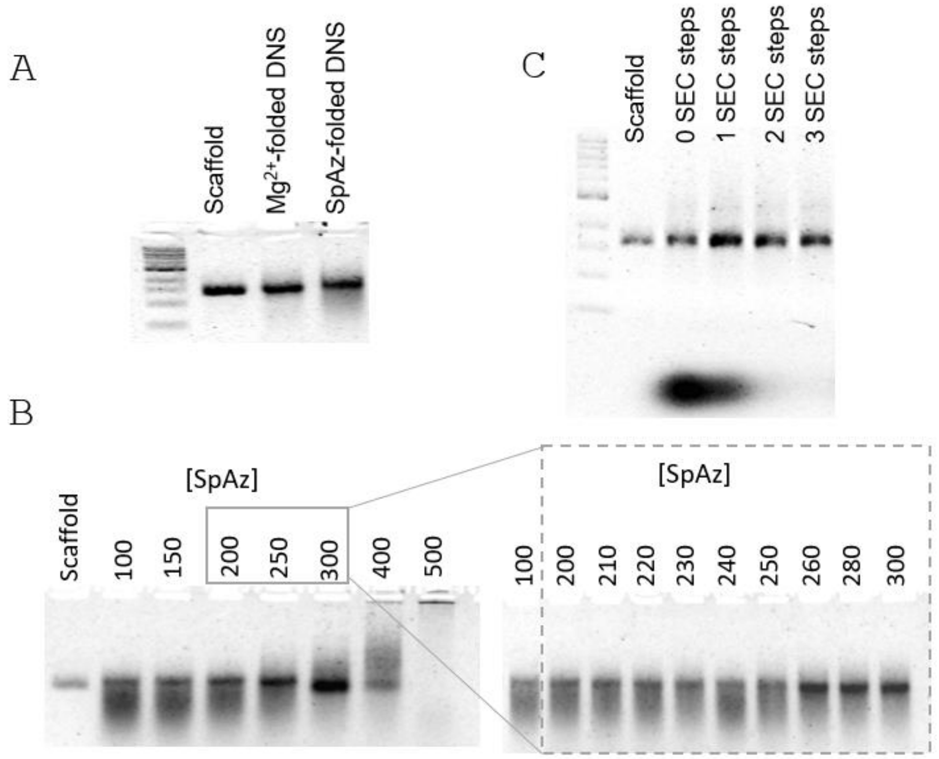

Figure S4. Agarose gel EMSA for origami DNS assembled with SpAz. A) Comparison between SpAz-folded DNS and a control Mg^{2+}-folded origami. B) DNS assembled with a range of SpAz concentrations (µM). Note that DNS are not adequately folded at SpAz concentrations below 250 µM and aggregation was dominant at 500 µM SpAz. C) Tracking of SEC purification process. Each step corresponds with a pass through a 600 µL resin-packed column.

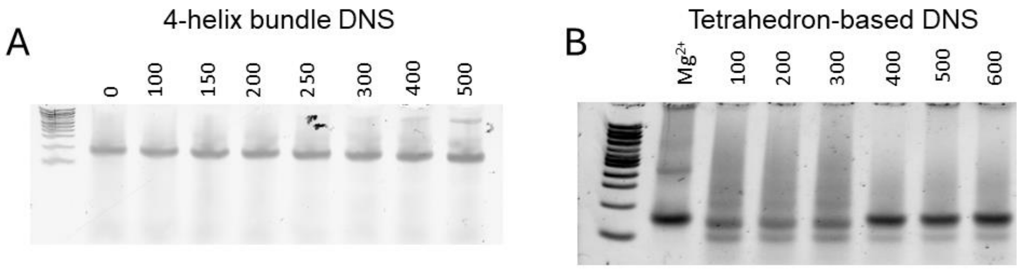

Figure S5. Polyacrylamide EMSA of A) 4-helix bundle and B) Tetrahedron-based DNS assembled with a range of SpAz concentrations (µM).

2.4. Cy5 molecules per DNS

Table S4. Data gathering the calculated concentration of Cy5 in samples after purification using the Beer-Lambert method and determination of the number of Cy5 molecules per DNS and base pairs (bps) per Cy5 on each Cy5-functionalized DNS.

DNS	bps	[DNS] (nM)	[Cy5] (µM)	Cy5/DNS	bps/Cy5
Origami-type	~7500	6.25	1.3	208.0	36.1
4-helix bundle	84	500	1.3	2.6	32.3
Tetrahedron	102	1000	2.9	2.9	35.2

2.5. Gel electrophoresis of Cy5. DBCO modified DNS

Cy5-modified DNS were analysed using GE (Figure S6) to demonstrate the integrity of the nanostructure as well as their fluorescence. Visualization of purified samples was performed with a ChemiDoc system, recording at both the GelRed and Cy5 channels.

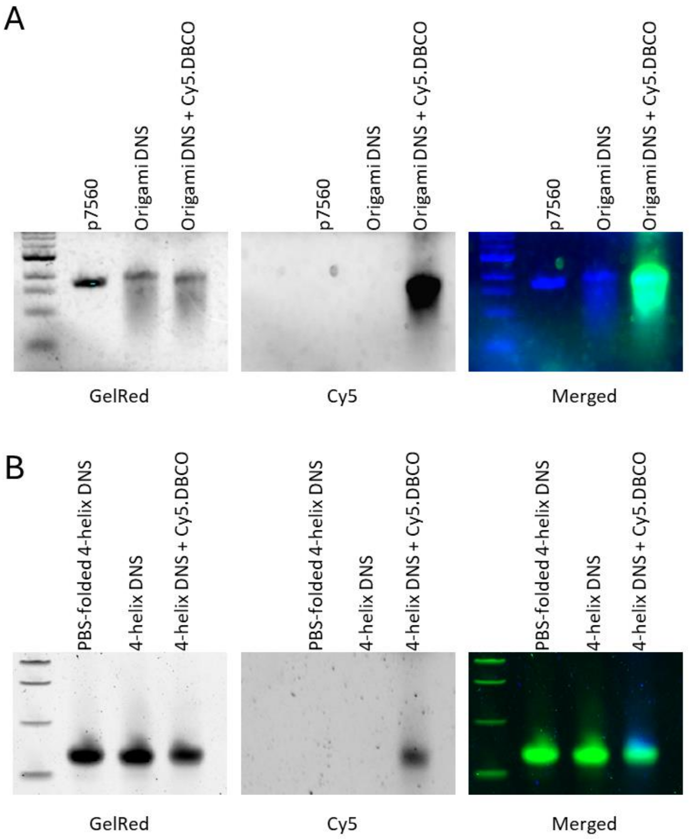

Figure S6. Agarose gel images of Cy5 modified DNS. A) Origami DNS. B) 4-helix bundle DNS. Each channel (GelRed and Cy5) is shown in separate columns.

2.6. Visualization of DNA origami with 48 Cy5 labels by confocal microscopy

To verify the high fluorescence observed in the SpAz-folded DNA origami functionalized with Cy5, a standard 48 Cy5-labelled origami sample was also analyzed using confocal microscopy. Notably, the laser power for the Cy5 channel was set to 0.5% in both the SpAz-folded DNA functionalized with Cy5 (Figure 3d main text) and the Mg^{2+}-folded DNA with 48 Cy5 labels to efficiently observe differences in fluorescence intensity (Figure S7A). Cy5 brightness seetings are enhanced in Figure S7B to probe colocalization with Hoechst channel.

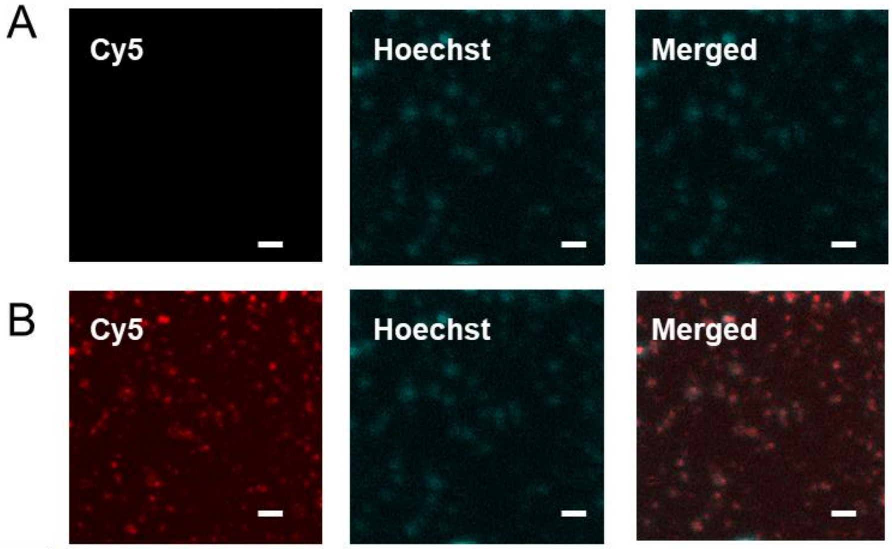

Figure S7. Confocal microscopy images of 48 Cy5-labeled DNA origami. Each channel (Cy5 and Hoechst) is displayed in separate columns. For the Cy5 channel, brightness settings are adjusted as follows: (A) identical to those used for imaging SpAz-folded DNA origami functionalized with Cy5 (Figure 3d, main text), and (B) increased by 10×. Scale bar corresponds to 2 μm.

2.7. Hydrodynamic diameter (Dh) values for PEG modified DNS

The Dh of SpAz-folded DNS functionalized with PEG 10kDa (as described in section 1.2.) was determined by DLS. To verify that size increase was produced by PEG modification, Sp-folded DNS reacted with PEG were also prepared and measured. For comparison, Dh values of Mg^{2+} folded DNS and SpAz-folded DNS are included (Table S5). Dh values for 4-helix DNS and tetrahedron-shaped DNS before and after PEG functionalization were also determined. Note that for 4-helix DNS, modification was carried out using a PEG.DBCO of 5 kDa due to the lower molecular weight to facilitate its subsequent removal by filtration with 10 kDa filters (Amicon). Reported values represent the average of 10 measurements, each comprising 5 reads per run.

Table S5. Dh values for DNS and controls in different folding buffers.

DNS	Dh (nm)				
	Mg	*SpAz*	*SpAz + PEG*	*Sp*	*Sp +PEG*
**Origami**	46.3 ± 2.2	47.2 ± 4.6	74.8 ± 8.4	44.6 ± 8.2	47.7 ± 6.7
**4-helix bundle**	7.1 ± 1.6	7.3 ± 0.5	11.6 ± 1.2	7.6 ± 0.8	5.5 ± 1.4
**Tetrahedron**	11.2 ± 0.6	9.7 ± 0.9	15.4 ± 1.3	10.5 ± 1.0	10.9 ± 0.7

2.8. AFM images and data

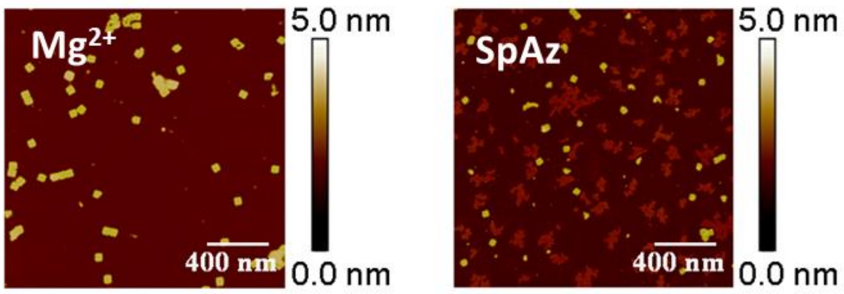

Figure S8. Representative AFM images of Mg^{2+}-folded DNS and SpAz-folded DNS. Scan size is 2 µm x 2 µm for all the images.

Several parameters including roughness, height, volume, and Young's modulus were estimated for Mg^{2+}-folded DNS, SpAz-folded DNS and SpAz-folded DNS modified with PEG.DBCO by AFM (Table S6). Box plots of all the collected AFM data with the statistical significance outcome are displayed in Figure S9. Note that SpAz-folded DNS exhibits greater compactness compared to Mg^{2+}-folded DNS. This may be attributed to the enhanced ability of multivalent cations, such as spermidine[16] or spermine[17] to strengthen DNA adsorption on mica. Figure S10 displays the roughness analysis on representative AFM topographic images.

Table S6. Compilation of the values (mean and SD) corresponding to the height, volume, roughness and Young's modulus parameters estimated for the DNA nanostructures measured by AFM. The data include measurements from N = 50 for height, volume, and roughness parameters, and N = 100 for Young's modulus.

Sample	Height (nm)	Volume (nm^3)	Roughness (Ra, nm)	Young's modulus (MPa)
Mg^{2+}-folded	4.7 ± 0.2	11920 ± 882	0.23 ± 0.04	576 ± 137
SpAz-folded	3.5 ± 0.2	7718 ± 877	0.23 ± 0.04	580 ± 198
SpAz-folded + PEG	5.0 ± 0.3	11978 ± 1860	0.33 ± 0.05	421 ± 128

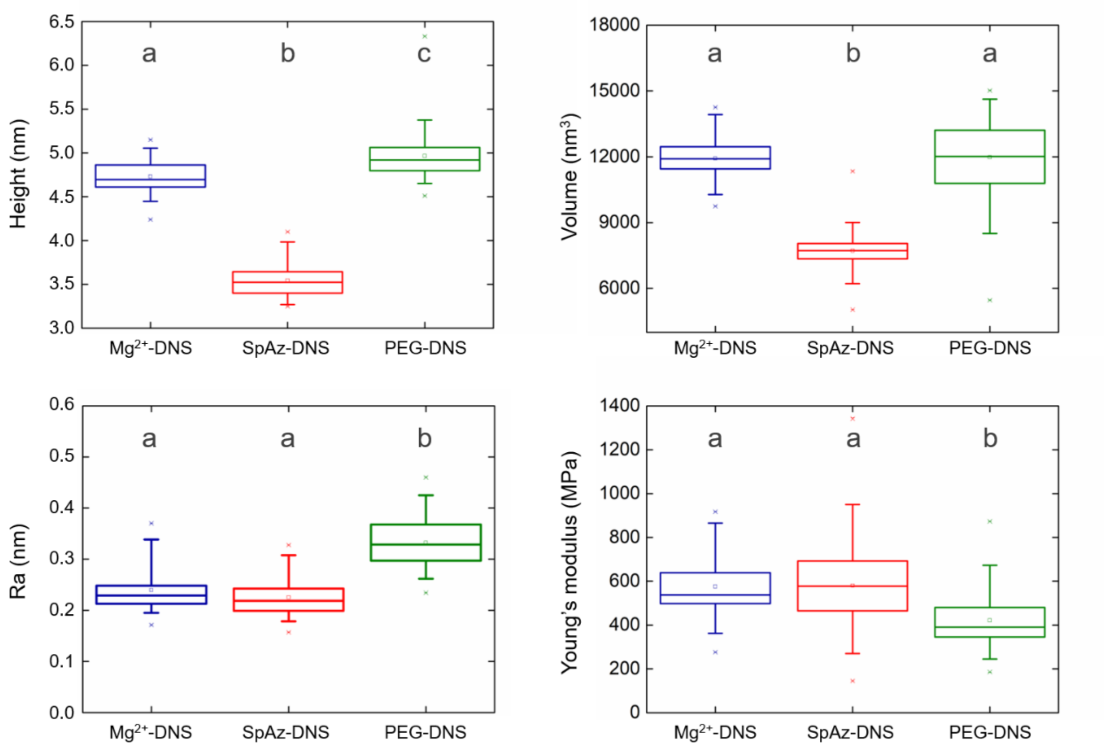

Figure S9. Statistical analysis of height, volume, roughness (Ra), and Young's modulus values using box plots for Mg^{2+}-folded DNS, SpAz-folded DNS, and PEG-functionalized DNS. Samples marked as a, b, and c exhibit statistically significant differences ($p < 0.05$) among each other ($a \neq b$, $a \neq c$, $b \neq c$).

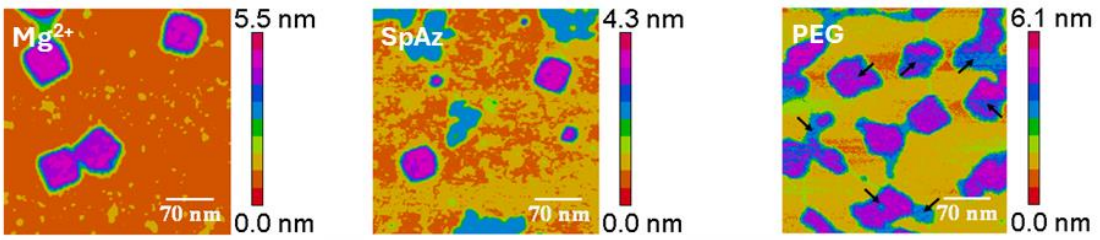

Figure S10. Roughness (Ra) analysis of Mg^{2+}-folded DNS, SpAz-folded DNS and SpAz-folded DNS modified with PEG.DBCO. Representative AFM topography images. Black arrows indicate the regions where the height strongly decays (blue colour) respect to the height of the top of the particles (in purple). This effect does not exist in the conditions depicted in DNS without PEG.DBCO. Scan size is 350 nm x 350 nm for all the images.

2.9. Transmission electron microscopy images

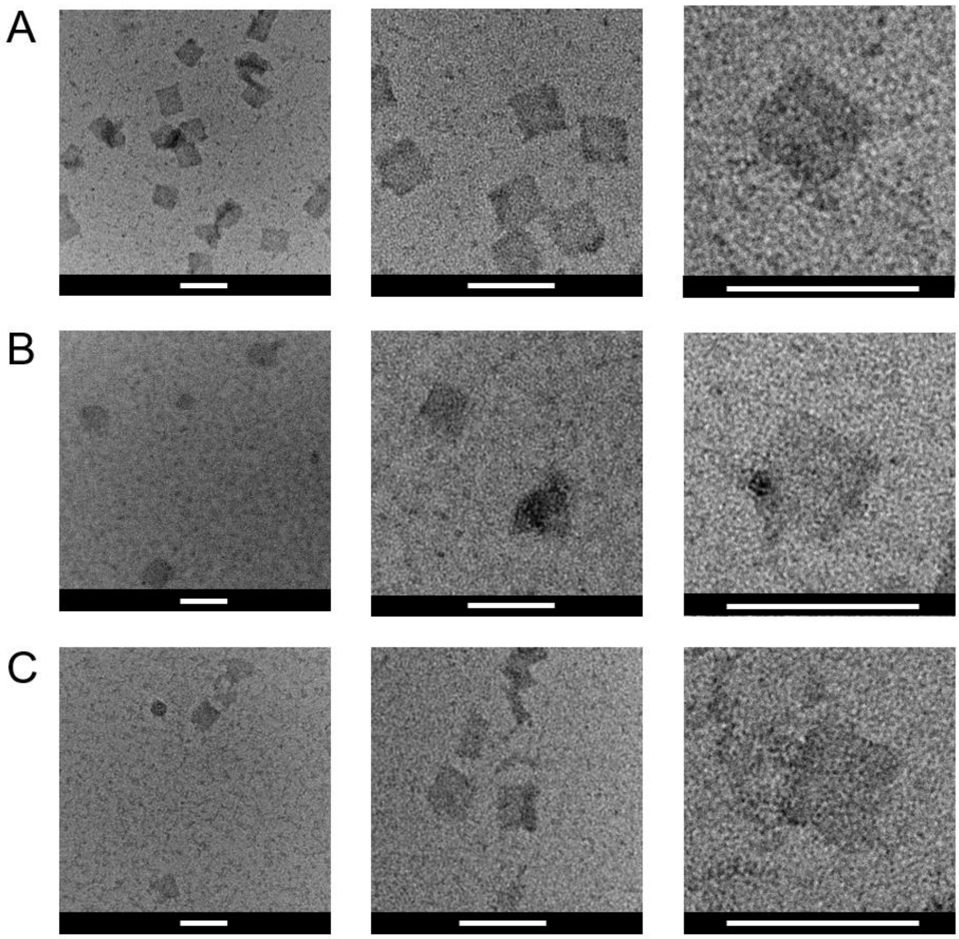

Figure S11. TEM images of DNS samples stained with uranyl acetate. A) Mg^{2+}-folded DNS, B) SpAz-folded DNS and C) PEG functionalized DNS. Scale bars correspond to 100 nm.

2.10. Attachment of DNS to GUVs containing PE.DBCO and controls

SpAz-folded DNS (containing 48 Cy5 labelled strands, Figure S3C) were incubated with GUVs containing PE.DBCO and observed by confocal microscopy to test the SPAAC mediated attachment (Figure S12). Controls GUVs without PE.DBCO were also observed (Figure S13). Additionally, confocal images for atto488-labeled tetrahedron-shaped DNS were taken (Figure S14 and S15). Furthermore, confocal images for SpAz-folded DNS fluorescently labelled (via SPAAC reaction with Cy5-DBCO) with GUVs are shown as Figures S16 and S17.

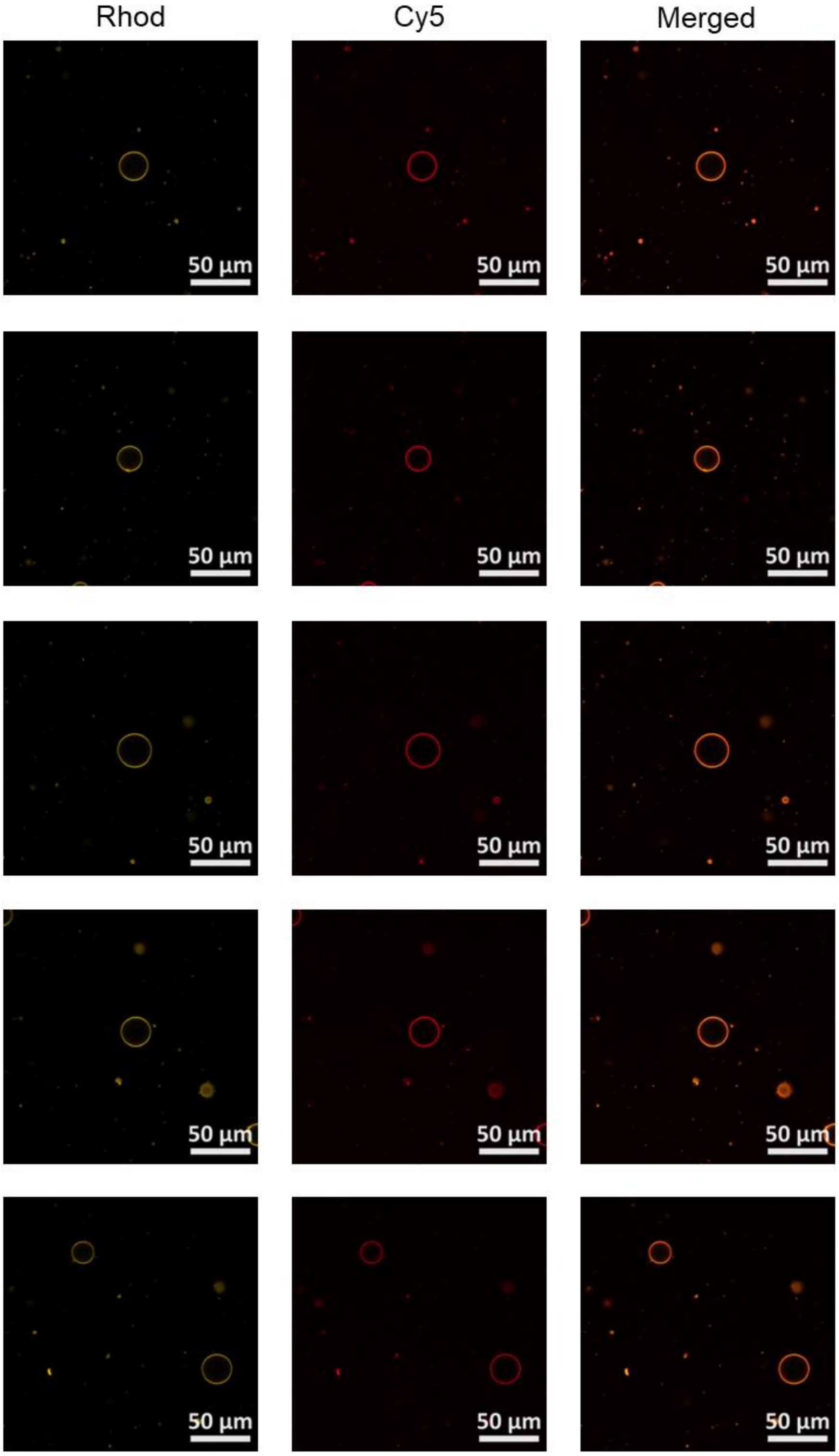

Figure S12. Complementary confocal images of GUVs with 5% PE.DBCO incubated with SpAz-folded origami (Cy5-labeled). Each channel (Rhodamine and Cy5) is shown in separate columns.

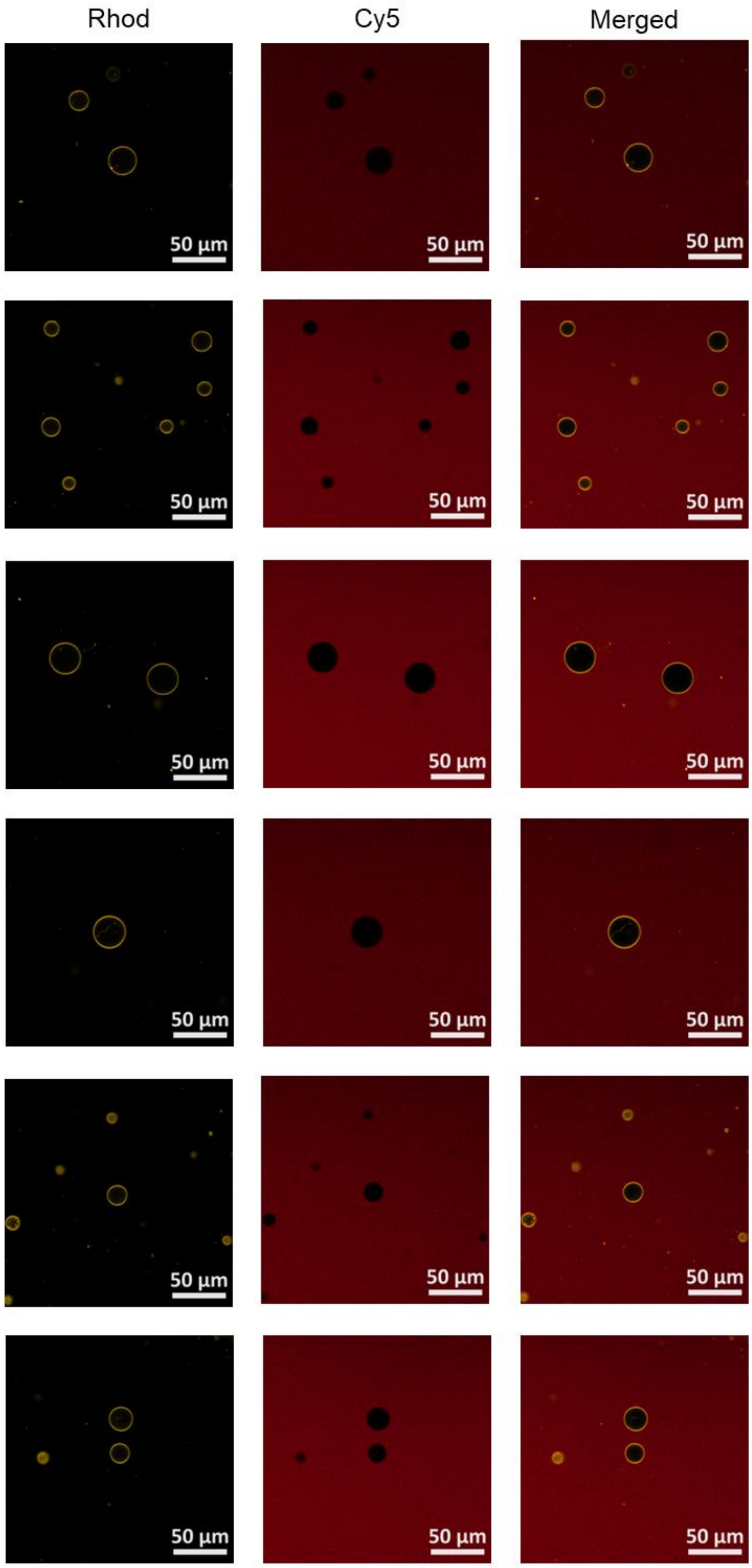

Figure S13. Complementary confocal images of GUVs without PE.DBCO incubated with SpAz-folded origami (Cy5-labeled). Each channel (Rhodamine and Cy5) is shown in separate columns.

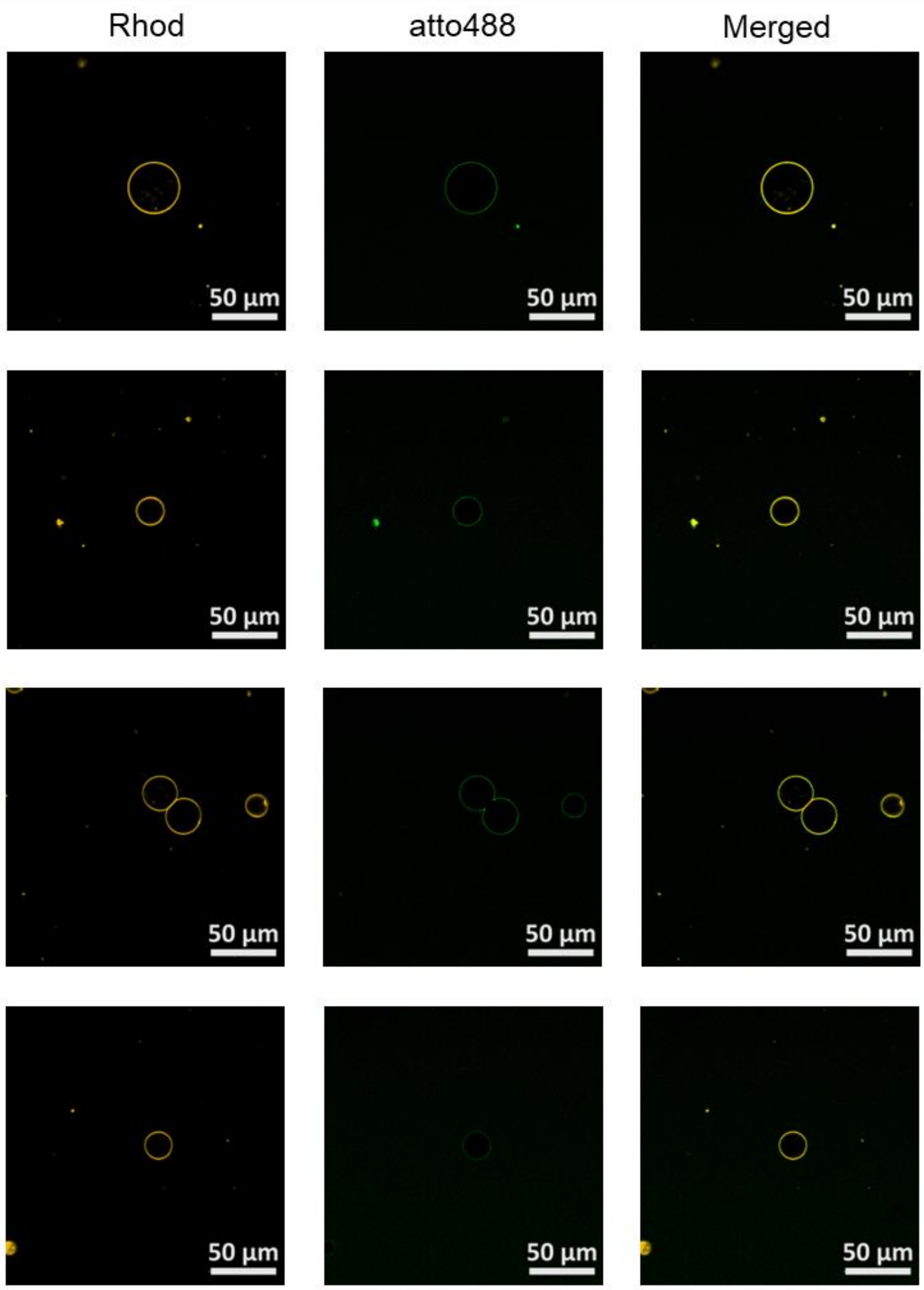

Figure S14. Confocal images of GUVs with 5% PE.DBCO incubated with SpAz-folded tetrahedron (atto488-labeled). Each channel (Rhodamine and atto488) is shown in separate columns.

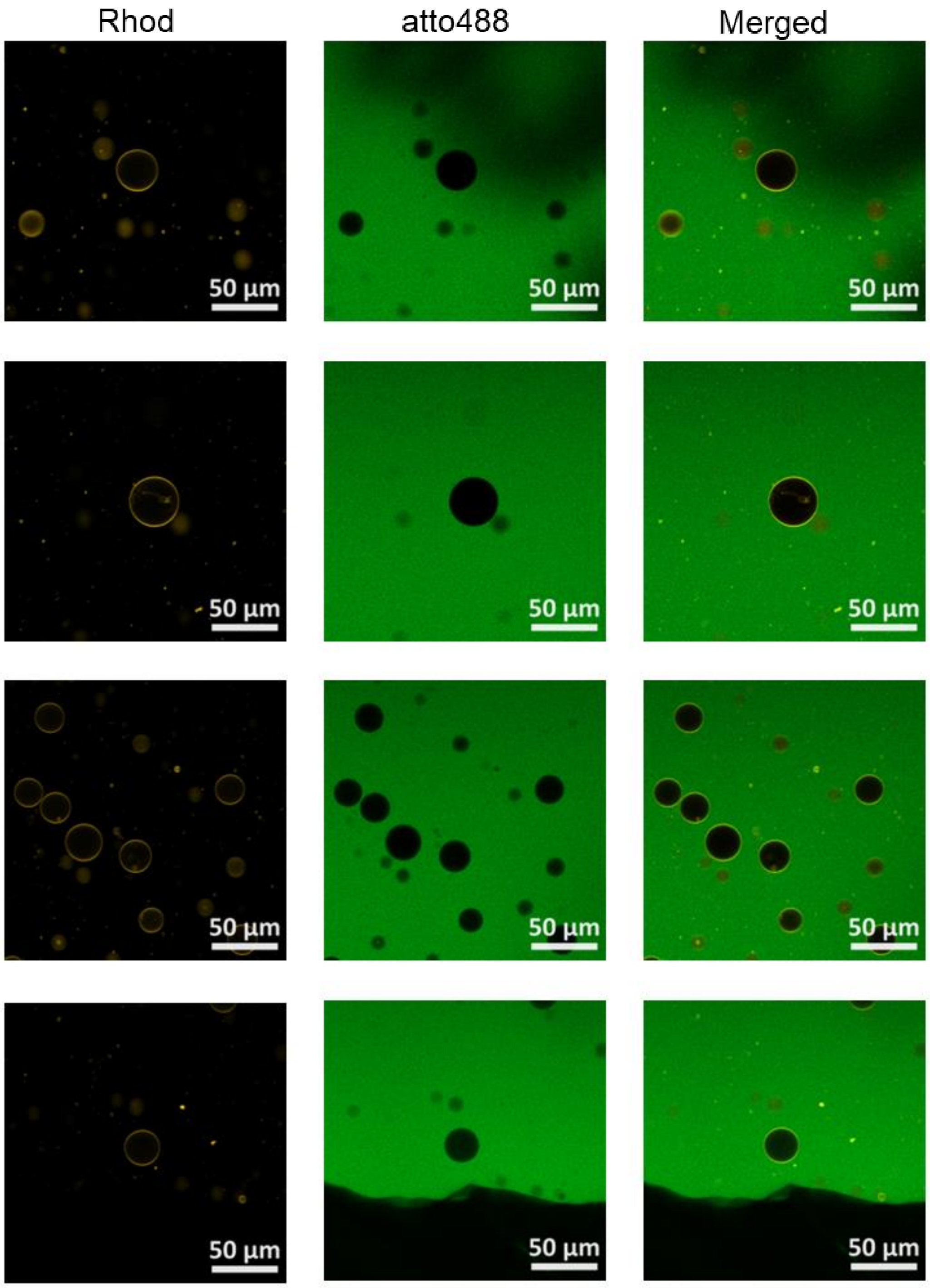

Figure S15. Confocal images of GUVs without PE.DBCO incubated with SpAz-folded tetrahedron (atto488-labeled). Each channel (Rhodamine and atto488) is shown in separate columns.

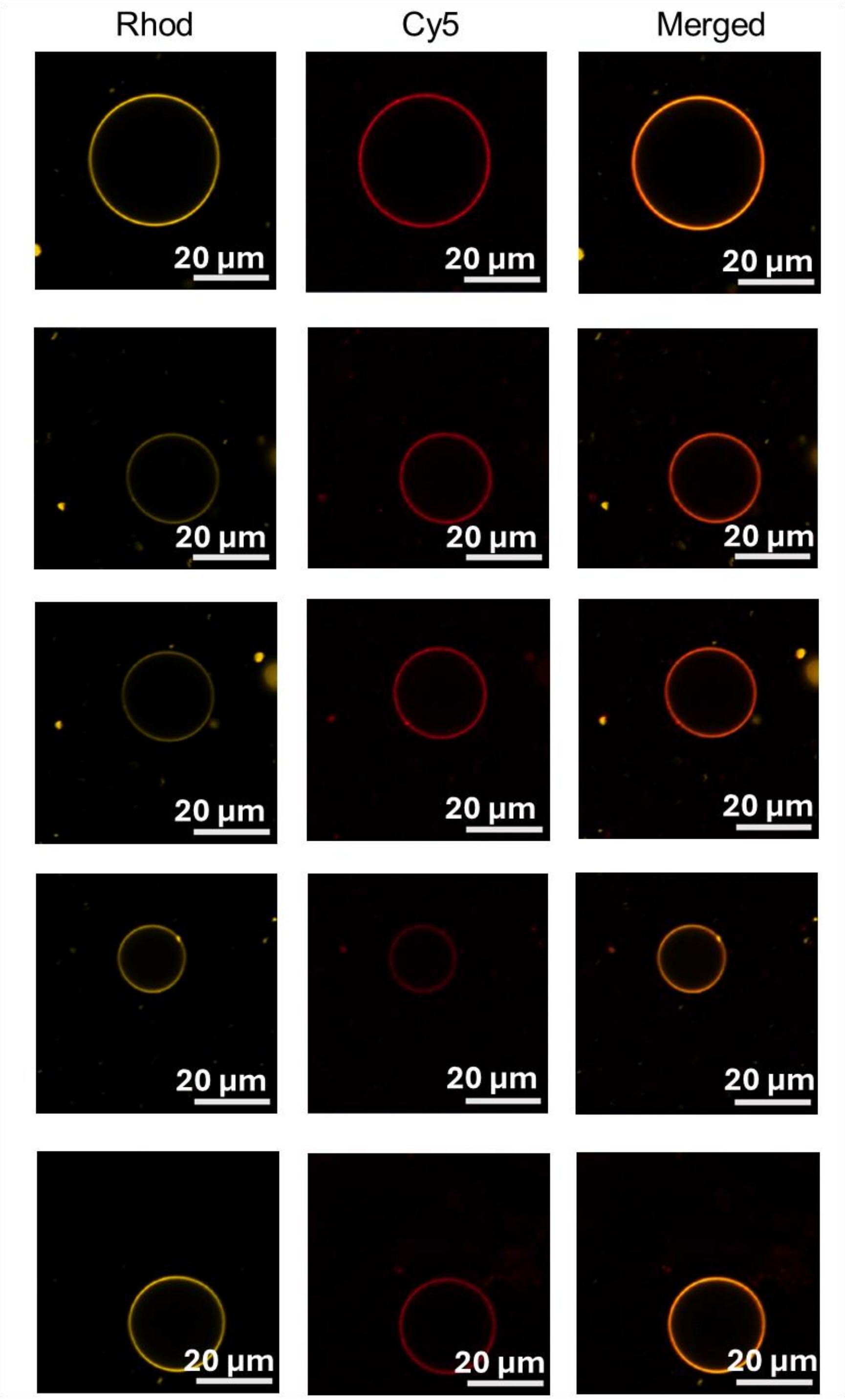

Figure S16. Confocal images of GUVs with 5% PE.DBCO incubated with SpAz-folded origami functionalized with Cy5-DBCO by SPAAC reaction. Each channel (Rhodamine and Cy5) is shown in separate columns.

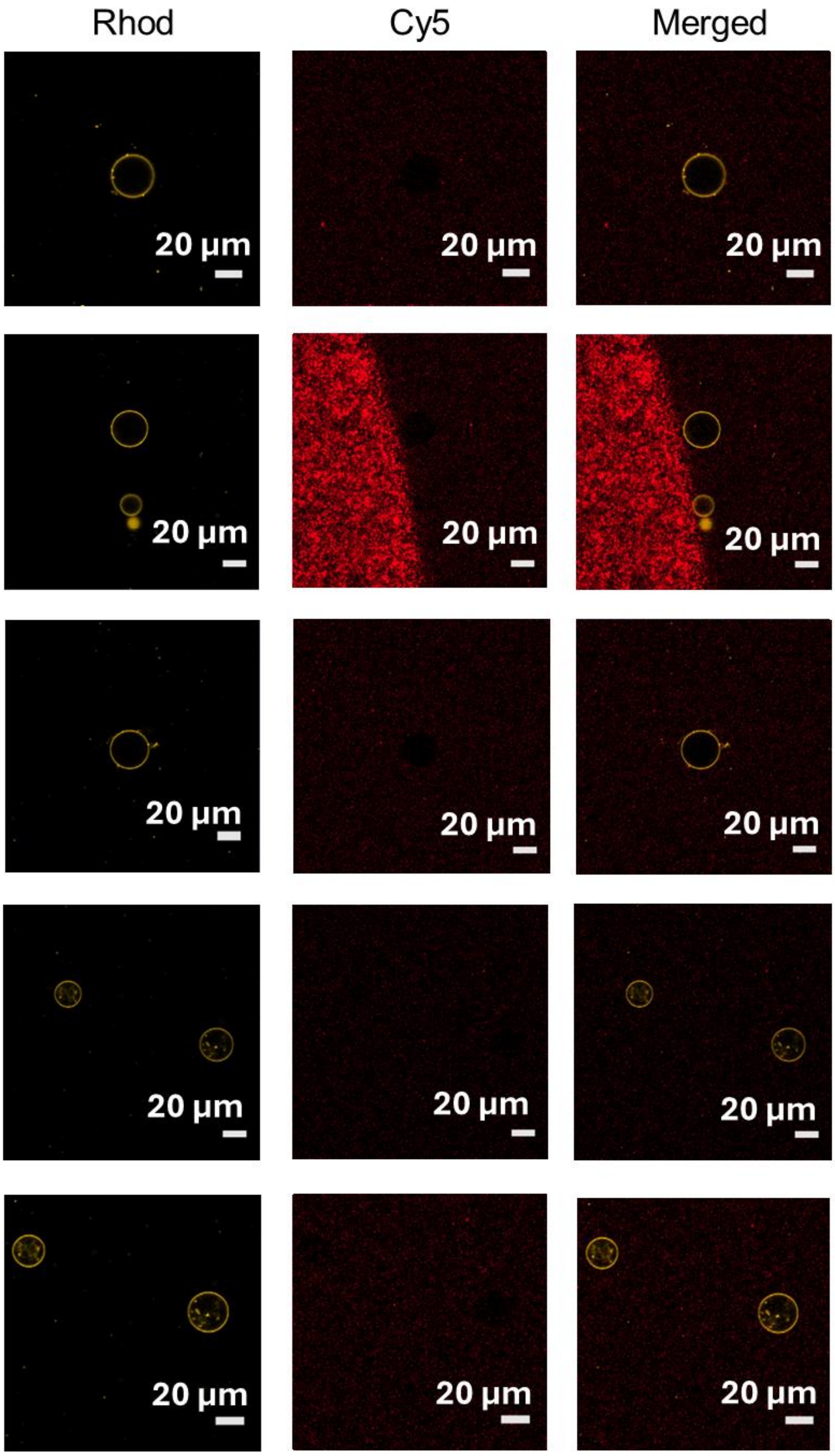

Figure S17. Confocal images of GUVs without PE.DBCO incubated with SpAz-folded origami functionalized with Cy5-DBCO by SPAAC reaction. Each channel (Rhodamine and Cy5) is shown in separate columns.

3. References

[1] C. Huang, Q. You, J. Xu, D. Wu, H. Chen, Y. Guo, J. Xu, M. Hu, H. Qian, Adv. Healthc. Mater. 2022, 11, 2200008.

[2] A. Postigo, P. Martínez-Vicente, K. N. Baumann, J. del Barrio, S. Hernández-Ainsa, Biomater. Sci. 2024, 12, 1549–1557.

[3] A. Chopra, S. Krishnan, F. C. Simmel, Nano Lett. 2016, 16, 6683–6690.

[4] L. Mallik, S. Dhakal, J.Nichols, J. Mahoney, A. M. Dosey, S. Jiang, R. K. Sunahara, G. Skiniotis, N. G. Walter. ACS Nano 2015, 9, 7, 7133–7141.

[5] K. Xu, W. Sun, Y. Shao, F. Wei, X. Zhang, W. Wang, P. Li, Nanotechnol. Rev. 2018, 7, 605–621.

[6] A. Lostao, K. Lim, M. C. Pallarés, A. Ptak, C. Marcuello, Int. J. Biol. Macromol. 2023, 238, 124089.

[7] A. Pyne, R. Thompson, C. Leung, D. Roy, B. W. Hoogenboom, Small 2014, 10, 3257–3261.

[8] K. Meinander, T. N. Jensen, S. B. Simonsen, S. Helveg, J. V Lauritsen, Nanotechnology 2012, 23, 405705.

[9] J. L. Hutter, J. Bechhoefer, Rev. Sci. Instrum. 1993, 64, 1868–1873.

[10] I. Horcas, R. Fernández, J. M. Gómez-Rodríguez, J. Colchero, J. Gómez-Herrero, A. M. Baro, Rev. Sci. Instrum. 2007, 78, 13705.

[11] D. Nečas, P. Klapetek, 2012, 10, 181–188.

[12] A. Magazzù, C. Marcuello, Nanomaterials 2023, 13, 963.

[13] B. V Derjaguin, V. M. Muller, Y. Toporov, J. Colloid Interface Sci. 1975, 53, 314–326.

[14] G. S. Manning, Soft Matter 2012, 8, 9334–9337.

[15] D. Morzy, R. Rubio-Sánchez, H. Joshi, A. Aksimentiev, L. Michele, U. Keyser, J. Am. Chem. Soc. 2021, 143.

[16] D. Pastré, L. Hamon, F. Landousy, I. Sorel, M- David, A. Zozime, E. Le Cam, O. Piétrement, Langmuir 2006, 22, 15, 6651–6660.

[17] K. Besteman, K. van Eijk, I. D. Vilfan, U. Ziese, S. G. Lemay. Biopolymers, 2007, 87, 141–148.
